# Topological characteristics and longitudinal dynamics of co-abundance networks involving beneficial commensal bacteria in the pig gut microbiome and its association with average daily gain

**DOI:** 10.3389/fmicb.2026.1818141

**Published:** 2026-04-29

**Authors:** Yuxin Liu, Congying Chen, Jun Gao

**Affiliations:** National Key Laboratory of Pig Genetic Improvement and Germplasm Innovation, Jiangxi Agricultural University, Nanchang, China

**Keywords:** average daily gain, beneficial commensal bacteria, co-abundance network, guild, pigs

## Abstract

Microorganisms are intricately interrelated with each other in the gut microecosystem, which influences the colonization and functional roles of probiotics. However, how these interactions dynamically change during host development and whether their topological features influence host phenotypes, such as average daily gain (ADG), remain poorly understood. In this study, we performed metagenome analysis for 2,311 fecal samples collected from a specifically designed eight genetically divergent breed intercrossed mosaic F6 and F7 population, at three developmental ages of 25 days (D25), 120 days (D120), and 240 days (D240) of each individual, covering pre-weaning to market. By constructing their microbiota co-abundance networks, we systematically characterized dynamic changes in beneficial commensal bacteria involved co-abundance networks in the pig gut microbiome across three ages. We elucidated conserved and variable co-abundance features involving these bacteria across developmental stages. We observed that the cross-age stable co-abundance correlations of beneficial commensal bacteria were maintained by a large set of weak correlations. A subset of age-shared co-abundance correlations remained variable across different ages in correlation strength and direction. Topological analysis revealed that beneficial commensal bacteria involved co-abundance networks were highly age-specific. Among the three age stages sampled in this study, the D120 stage represented a critical window for the structural and functional reorganization of gut microbiota. Using metagenomic sequencing data at the D120, we identified two guilds that were significantly associated with ADG from D120 to D240. Guild 1 included short chain fatty acid-producing taxa and was positively associated with ADG, whereas Guild 2 tended to self-utilization of energy and was negatively associated with ADG. We also inferred the ecological interaction mechanisms of ADG-associated microbial communities using genome-scale metabolic models. These findings provided a theoretical basis for stage-specific intervention in the pig gut microbiome using probiotics to improve production traits.

## 1 Introduction

Pigs are important agricultural animals and represent a major source of meat for human consumption. There are approximately 10^13^∼10^14^ bacteria in an adult pig’s gastrointestinal tract, and their gene count greatly exceeds the number of the host’s own genes. Therefore, the gut microbial genomes are regarded as the host’s “second genome” (Sender et al., 2016; Huang and Chen, 2023; O’Hara and Shanahan, 2006). The gut microbial community plays critical roles in host physiological processes, including nutrient metabolism, immune regulation, and disease defense (de Vos et al., 2022). In recent years, with the widespread application of omics technologies, growing evidences have demonstrated that, in addition to significant effects on host nutrition and disease status, the gut microbiota also significantly influences production traits in farm animals. In pig production, gut probiotics have shown considerable potential as feed additives, providing a convenient and effective strategy for improving production performance, and also contribute to the sustainable development of agricultural animals amid reductions and bans on antibiotic use. Numerous studies have demonstrated that the supplementation with specific probiotics can significantly improve growth performance (Bergamaschi et al., 2020; Luo et al., 2021) and meat quality (Lian et al., 2024; Yin et al., 2023) in pigs. For example, the supplementation with gut-derived probiotics, including *L. johnsonii*, *L. mucosae*, and *L. plantarum*, has increased growth rate and feed efficiency in weaned piglets and growing-finishing pigs, while simultaneously reducing the abundance of potential pathogenic *E. coli* (Thu et al., 2011; Giang et al., 2011). Ramayo-Caldas et al. identified two enterotypes with distinct metabolic potentials. The carbohydrate fermentation-associated enterotype was associated with higher ADG, while the cellulose- and lignin-degrading enterotype showed lower ADG (Ramayo-Caldas et al., 2016). The beneficial effects of probiotics are mainly mediated by a range of metabolites and cell components produced during their colonization in the gastrointestinal tract, including short-chain fatty acids (SCFAs) (Mukhopadhya and Louis, 2025), bacteriocins (Heilbronner et al., 2021), antimicrobial peptides (Yu et al., 2026), and other microbial metabolites. These metabolic products contribute to the regulation of intestinal barrier function and immune status, alleviate inflammation, and thereby promote host health (Ma et al., 2023). However, many probiotics exhibit limited capacity for long-term, stable colonization following ingestion, and their beneficial effects often depend on interactions with other commensal bacteria in the same ecological niche (Jiang et al., 2025).

The gut microbiome functions as a complex adaptive ecosystem (CAS), in which community composition and ecosystem-level traits are jointly shaped by interactions among its members (Bakkeren et al., 2025). These ecological interactions govern microbial community stability and resilience, and they give rise to system-level emergent properties that extend beyond the characteristics of individual taxa (Wu et al., 2024). Within this ecological framework, gut microbes with similar functions often coexist as guilds. Members in the same guild may derive from distinct phylogenetic lineages. However, under specific physiological or environmental conditions, they frequently exhibit consistent co-abundant behavior. This pattern may reflect shared strategies of resource utilization or the presence of certain interactive molecular mechanisms (Wu et al., 2021).

Although numerous studies have reported associations between gut microbial taxa and host phenotypes, most have focused on individual microbes, overlooking interactions among microbial community members. It has been shown that *L. citreum* enhances the colonization ability of *L. plantarum* by synergistically modifying the intestinal niche (Jiang et al., 2025). The interaction networks between beneficial commensal bacteria and other bacterial species in the gut microbial community, and their dynamic changes across developmental stages, have received less attention and remain poorly understood. The common and age-specific interaction networks between beneficial commensal bacteria and other bacteria across the developmental stages remain unexplored. Previous studies have suggested that the alterations in gut microbiota structure and functions during critical developmental windows can have lasting effects on host growth and metabolism (Warne et al., 2019). However, the ecological mechanisms underlying these effects remain unclear. We either do not know whether the changes in network topologies between beneficial commensal bacteria and other bacteria with ages will affect host health and growth. From a system ecology perspective, elucidating gut microbiota network dynamics and their ecological mechanisms across distinct host growth stages is essential for understanding the principles governing microbial community assembly during host development and its potential effects on host phenotypes.

In this study, to improve the colonization efficiency of probiotics in the pig gut, we first performed an investigation of the co-abundance interaction networks of beneficial commensal bacteria with gut microbes using a large-scale 2,311 metagenomic sequencing data from F6 and F7 generation pigs of a mosaic pig population, which were collected fecal samples at 25, 120, and 240 days of age. And then, we characterized the dynamic changes in beneficial commensal bacteria involved gut microbial interaction networks with age. We identified conserved and age-stage-specific interaction patterns of beneficial commensal bacteria involved co-abundance networks. We finally identified average daily gain (ADG)-associated microbes at the strain level. The ecological mechanisms underlying these ADG-associated microbes were then examined across multiple dimensions, including co-abundance characteristics, functional differentiation, and metabolic interaction patterns. By elucidating the dynamic changes in beneficial commensal bacteria involved co-abundance networks, the results may contribute to improving probiotic colonization in interventions targeting the pig gut microbiome at specific age stages to increase pig growth speed.

## 2 Materials and methods

### 2.1 Experimental animals and sample collection

This study used two well-defined experimental pig populations of F6 and F7 derived from a mosaic population (Yang H. et al., 2022). The mosaic population was constructed using eight founder pig breeds of four Western commercial breeds (Duroc, Landrace, Large White, and Pietrain) and four Chinese indigenous breeds (Bama Xiang, Erhualian, Laiwu, and Tibetan pigs) through a series of intercrosses, beginning with the F0 generation, in which boars and sows from Chinese indigenous breeds were mated with those from different Western commercial breeds. Over several generations (F1–F3), the populations were gradually intercrossed to increase genetic diversity, resulting in highly recombinant lines by the F6 and F7 generations (Yang H. et al., 2022). All pigs were raised under uniform housing and management conditions and remained in good health, with no antibiotic treatment administered for at least 2 months prior to sample collection. Animals were fed commercial formula diets according to standard nutritional requirements. Pigs at 25, 120, and 240 days of age were provided with diets containing different nutrient levels as described previously (Ke et al., 2019). The detailed components of formula diets are provided in Supplementary Table S1.

Fecal samples were collected at three ages: day 25 (the suckling period), day 120 (growing period), and day 240 (slaughter). A total of 1,166 fecal samples were collected from the F6 population, including 140 samples at the age of day 25, 393 samples at the age of day 120, and 633 at day 240. For the F7 population, a total of 1,145 fecal samples were collected, including 169, 413, and 563 feces samples at the ages of 25, 120, and 240 days, respectively. All samples were immediately snap-frozen in liquid nitrogen and stored at -80 °C until further processing. All animal-related experimental procedures were approved by the Ethics Committee of Jiangxi Agricultural University (approval number JXAU2011-006).

### 2.2 Measurement of ADG related phenotypes

Body weights were recorded at 120 and 240 days. In the F6 population, 265 pigs had body weight data at both time points. Average daily gain (ADG) was calculated as the difference in body weight between D240 and D120 divided by the number of days between the two measurements. Pigs with extreme ADG values were selected and classified into two groups: a high ADG group (HADG, *n* = 28) and a low ADG group (LADG, *n* = 27). The two groups did not differ significantly in body weight at 120 days, minimizing the potential influence of initial body weight on ADG comparisons.

### 2.3 Metagenomic sequencing and bioinformatic analysis

Microbial DNA from fecal samples was extracted using the E.Z.N.A Soil Microbe DNA Kit (M9636-02, Omega Bio-Tek, United States) following the manufacturer’s guidelines. Libraries with an insert size of approximately 400 bp were constructed and sequenced on an Illumina NovaSeq 6000 platform, generating 150 bp paired-end reads. Except 311 feces samples that were sequenced in our previous study (Chen et al., 2021), all other samples were sequenced in this study. Raw reads were quality-controlled using fastp (v0.20.0) (Chen, 2023), including adapter trimming, filtering low-quality reads, and removing reads shorter than 60 bp. Host genomic DNA contamination was removed by aligning filtered reads to the pig reference genome (Sus scrofa 11.1) using BWA-MEM2 (v2.2.1) (v2.2.1) (Vasimuddin et al., 2019), followed by processing with SAMtools (Danecek et al., 2020). De novo assembly was performed for each sample using MEGAHIT (v1.1.3) (Li et al., 2014). Contigs were further binned separately using MetaBAT2 (v2.12.1) (Kang et al., 2019) and VAMB (v3.0.2) (Nissen et al., 2021). The quality of the bins was assessed using CheckM (v1.1.3) (Parks et al., 2015). Bins with completeness > 50% and contamination < 5% were retained as medium- and high-quality draft genomes, whereas those with completeness > 90% and contamination < 5% were defined as high-quality genomes (HQMAGs). All MAGs were dereplicated using dRep (v2.6.2) (Olm et al., 2017). MAG abundances were calculated using metaWRAP (v1.3) (Uritskiy et al., 2018). Taxonomic assignment of MAGs was performed using GTDB-Tk (v2.1.0) (Chaumeil et al., 2019). Protein-coding genes were predicted from MAGs using Prokka (v1.14.5) (Seemann, 2014), and functional annotation was performed using eggNOG-mapper (v2.1.3) (Cantalapiedra et al., 2021), KofamScan (v1.3.0) (Aramaki et al., 2019), dbCAN2 (v2.0.11) (Zhang et al., 2018), and gutSMASH (v.1.0) (Andreu et al., 2023).

### 2.4 Construction of beneficial commensal bacteria involved co-abundance networks

For the F6 and F7 populations, whole co-abundance networks were constructed at each age stage based on bacterial species present in at least 10% of samples. Microbial interaction networks were inferred using FastSpar (v1.0.0) (Watts et al., 2019) with 1,000 permutations, and only correlations with FDR < 0.05 were retained. Subnetworks involving beneficial commensal bacteria were extracted from the whole co-abundance networks at D25, D120, and D240 using the microeco package (v1.11.0) (Liu et al., 2026) in R (v4.0.3).

Beneficial commensal bacteria were defined as those gut commensal bacterial taxa which have been reported to show beneficial effects on hosts and/or recognized as candidate next-generation probiotics by multiple literatures. The taxa included were grouped into three categories: (1) lactobacilli and bifidobacteria that have been widely studied in probiotic-related researches (Mejía-Caballero and Marco, 2026), including *Lactobacillus* (Dempsey and Corr, 2022; Yang S. et al., 2022), *Ligilactobacillus* (Gilliland, 1990; Masood et al., 2011), *Limosilactobacillus* (Gul and Durante-Mangoni, 2024; Gilliland, 1990; Masood et al., 2011), *Weissella* (Teixeira et al., 2021), and *Bifidobacterium* (Gomes and Malcata, 1999; Gul and Durante-Mangoni, 2024); (2) genera widely discussed as candidate next-generation probiotics, such as *Akkermansia* (Cani et al., 2022; Panzetta and Valdivia, 2024) and *Christensenella* (Sun et al., 2024); and (3) short-chain fatty acid-producing genera with recognized beneficial effects, including *Blautia* (Liu et al., 2021), *Butyrivibrio* (Srivastava et al., 2021; Singh et al., 2023), *Coprococcus* (Azcarate-Peril et al., 2021; Rampelotto et al., 2025), *Eubacterium* (Mukherjee et al., 2020), and *Roseburia* (Tamanai-Shacoori et al., 2017). For those taxa whose beneficial effects have been mostly reported at the species level rather than at the genus level, we only retained those species, such as *Anaerobutyricum soehngenii* (Lee et al., 2024; Wortelboer et al., 2022) and *Faecalibacterium prausnitzii* (He et al., 2021; Bui and de Vos, 2021). The detailed information about selected taxa, the selection criteria applied, and corresponding supporting references has been added to Supplementary Table 2. The networks were visualized with Cytoscape v3.10.2.

### 2.5 Conservation and variability of co-abundance correlations across age stages in the beneficial commensal bacteria involved networks

Differences in beneficial commensal bacteria involved co-abundance networks across age stages were assessed using the metagen() function from the meta package (v8.0.1) in R (v4.0.3), and the correlation strength of each co-abundance correlation was compared among age groups using Cochran’s Q test. Co-abundance correlations with *P* > 0.05 in Cochran’s Q test were considered stable and conserved correlations. Correlations with I^2^ value > 75% and FDR < 0.05 were considered heterogeneous, reflecting variability across age stages.

### 2.6 Topological analysis of beneficial commensal bacteria involved co-abundance networks

Within beneficial commensal bacteria involved co-abundance networks, only co-abundance correlations with *r* > 0.2 were retained for topological analyses across age stages. Network topological features at three age stages were calculated using the microeco package (v1.11.0) in R (v4.0.3), including the number of nodes, number of edges, average degree, clustering coefficient, centralization, and modularity (Liu et al., 2026). Nodes were classified based on their within-module and among-module connectivity. Nodes identified as module hubs (Zi > 2.5 and Pi < 0.62), connectors (Zi > 2.5 and Pi > 0.62), or network hubs were defined as hub nodes within the co-abundance network. Nodes with Zi < 2.5 and Pi < 0.62 were classified as peripheral and regarded as non-hub nodes.

### 2.7 Assessment of stability, vulnerability, and robustness of beneficial commensal bacteria involved co-abundance networks

The influence of each node in beneficial commensal bacteria involved co-abundance networks across age stages was assessed using the abundance-weighted mean interaction strength (wMISi) index, as previously described (Li et al., 2023). Nodes shared across co-abundance networks at all three age stages were defined as core nodes. Network stability was calculated based on wMISi, the number of core nodes, and the total node count.

Node vulnerability was defined as the loss of network efficiency upon the removal of a node and its connected edges, reflecting the node’s contribution to network efficiency. The maximum node vulnerability was used to represent the entire network vulnerability, which was calculated at three age stages using the vulnerability function in the microeco package (v1.11.0) (Liu et al., 2026) in R.

Network robustness was defined as the ability of a network to maintain its structure under random or targeted node removal. To evaluate the impact of hub bacteria on network robustness, two removal strategies were applied: hub bacteria-based TR and non-hub bacteria-based RA, following previously described methods. In the TR simulation, half of the hub bacteria were randomly removed from the network, and the ratio of total wMISi after removal to the original total wMISi was calculated. An equal number of non-hub bacteria were randomly removed, and the resulting wMISi ratio was calculated using the same procedure. The process was repeated 10 times. After that, network robustness was compared between the TR and RA methods.

### 2.8 Construction of genome-scale metabolic models associated with ADG

GEMs were constructed for each HQMAG significantly associated with ADG using gapseq (v1.4.0) under default settings (Zimmermann et al., 2021). Metabolic competition and complementarity indices among HQMAGs were calculated from the GEMs using PhyloMint (v0.1.0). A combined metabolic distance score was defined as 1 – (competition index - complementarity index). Given that the metabolic competition and complementarity indices were negatively correlated, their Z scores were combined to generate a composite competition intensity metric defined as Z(metabolic competition index) - Z(metabolic complementarity index). This metric was used to identify competitive interactions between guild 1 and guild 2. Strain pairs in the upper quartile were classified as competitive, reflecting high resource overlap and low metabolic complementarity. Parsimonious flux balance analysis (pFBA) was conducted for each GEM to estimate flux distributions, and predicted exchange metabolites, including both secreted and uptake compounds, were derived. The metabolic potential of each HQMAG was assessed based on pathway annotations from the MetaCyc database.

### 2.9 Statistical analysis

All statistical analysis was performed in the R (v4.0.3). Bray-Curtis distances were computed using the vegdist() function in the vegan package (v2.5.7), and differences in gut microbiota structure across age stages were assessed using PERMANOVA with 999 permutations. Similarity among modules within whole-co-abundance networks of gut microbiota across age stages, and between these modules and beneficial commensal bacteria involved subnetworks extracted from the overall microbial co-abundance networks, was quantified using the Jaccard index, calculated as the size of the intersection divided by the size of the union of their members.

HQMAGs associated with ADG were identified using linear mixed-effects models implemented in MaAsLin2 (v1.15.1) (Mallick et al., 2021), with an adjusted *P* value cutoff of 0.2. Co-abundance correlations among genomes associated with ADG were calculated using FastSpar (v1.0.0) (Watts et al., 2019), and only correlations with *P* < 0.05 were retained for further analysis. KEGG Ortholog (KO) diversity in each guild was calculated from KOFam results using the Shannon index implemented in the vegan package (v2.5.7) in R. KOs significantly associated with either Guild 1 or Guild 2 were identified using generalized linear models. The proportions of primary metabolic gene clusters identified by gutSMASH (v1.0.0) were compared between Guild 1 and Guild 2 using Fisher’s exact test. In all statistical analyses, *P* values were adjusted for multiple tests, and significance was defined as FDR < 5%. Based on flux predictions, metabolites predicted to be secreted or uptake by HQMAGs in Guild 1 and Guild 2 were analyzed using a generalized linear model, as described above, to identify candidate metabolites.

## 3 Results

### 3.1 Dynamic changes in beneficial commensal bacteria involved co-abundance networks following the ages

**FIGURE 1:**
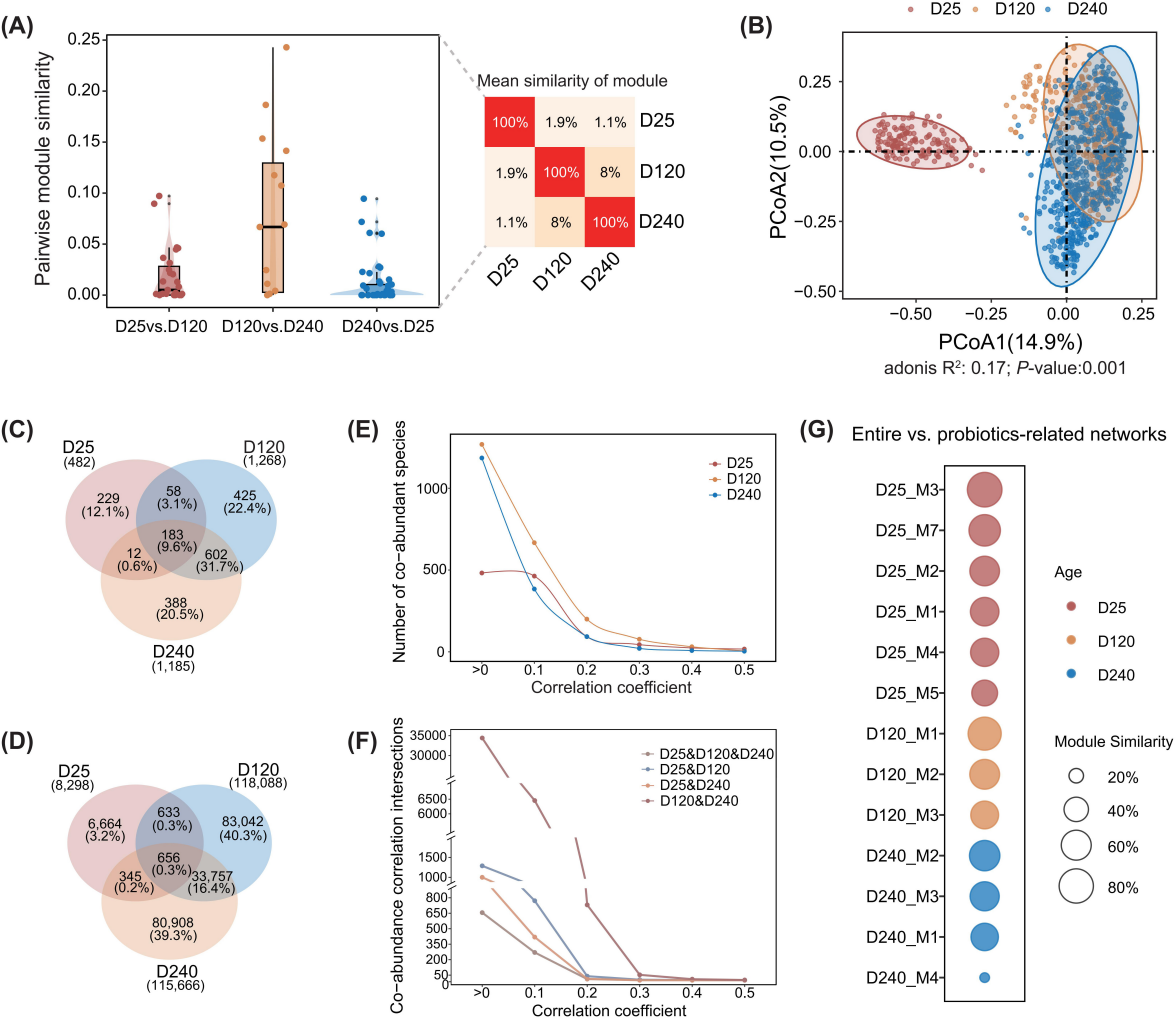
Characteristics of co-abundance networks involving beneficial commensal bacteria in the gut microbiota across three age stages in the F6 population. **(A)** Module similarity of gut microbial co-abundance networks between pairwise age stages. The left panel shows boxplots of similarity coefficients derived from pairwise comparisons of modules between pairwise age stages, and the right panel shows a heatmap of mean module similarity across the three age stages. **(B)** Principal coordinates analysis (PCoA) based on Bray-Curtis distances illustrating differences in gut microbial community composition at D25, D120, and D240. PERMANOVA was performed using 999 permutations, with Benjamini-Hochberg adjusted *P* < 0.05. **(C,D)** Numbers of shared and specific co-abundance correlations and the corresponding species in co-abundance networks involving beneficial commensal bacteria at D25, D120, and D240, including the number of species involved in co-abundance correlations **(C)** and the number of co-abundance correlations identified **(D)**. **(E,F)** Changes in co-abundance patterns with increasing correlation coefficients in the F6 population. **(E)** The number of species involved in co-abundance correlations, and **(F)** the number of co-abundance correlations shared among different age-stages following the correlation coefficients. The x-axis represents the co-abundance correlation coefficients. **(G)** Inter-module similarity between the whole gut bacterial co-abundance network and the subnetwork involving beneficial commensal bacteria in the F6 population. Bubble plots illustrate the similarity between modules of the beneficial commensal bacteria involved co-abundance subnetwork and modules of the whole bacterial co-abundance network at D25, D120, and D240. Bubble size indicates the values of inter-module similarity, and color represents the age stage.

Given the complex synergistic and antagonistic interactions among members of gut microbial communities, it is essential first to understand the interactions between beneficial commensal bacteria and other microbes in the community to improve probiotic colonization in the gut. We focused on beneficial commensal bacteria involved co-abundance networks by extracting them from the whole-co-abundance networks of the gut microbiota. We investigated the dynamic changes in beneficial commensal bacteria involved co-abundance networks from 25 days of age (D25, suckling period) to 120 (D120, fast growing period), and 240 days of age (D240, slaughter day) by using fecal metagenomic sequencing data from F6 and F7 pigs of a mosaic population. Bacterial species present in at least 10% of samples at each age stage were included in the construction of the whole-co-abundance networks. Gut microbial co-abundance networks were subsequently constructed for each population and age stage, resulting in six co-abundance networks in this study. Based on the values of the module similarity, the co-abundance network structure of the pig gut microbiota differed significantly among three age stages (D25, D120, and D240) in the F6 population (Figure 1A). This pattern was consistent with differences observed in microbial community composition (Figure 1B). A similar pattern was observed in the F7 population (Supplementary Figures S1A,B).

We then extracted subnetworks associated with beneficial commensal bacteria from the whole-microbial co-abundance networks at each age stage. In the F6 population, subnetworks at D25, D120, and D240 contained 53, 78, and 85 beneficial commensal bacteria species, respectively. These beneficial commensal bacteria species were from 10, 13, and 11 genera, respectively (see Methods). These subnetworks comprised of 482, 1,268, and 1,185 nodes and involved 8,298, 118,088, and 115,666 significant co-abundance correlations, respectively (FDR < 0.05) (Figures 1C,D).

The distribution of correlation coefficients for co-abundance correlations in beneficial commensal bacteria involved co-abundance networks differed across age stages (Figure 1E). Following the increase in correlation coefficient values, the number of co-abundance correlations shared across age stages decreased dramatically, suggesting that strong co-abundance correlations exhibit age-specific characteristics (Figure 1F). In addition, high module similarity was detected between beneficial commensal bacteria involved co-abundance networks and the whole microbial co-abundance networks at all three age stages, indicating that interaction patterns between beneficial commensal bacteria and other microbes within the gut ecosystem were largely consistent with those at the whole-community level (Figure 1G). Similar results were observed in the F7 population (Supplementary Figures S1C–G).

### 3.2 Conservation of beneficial commensal bacteria involved co-abundance networks across three developmental stages

**FIGURE 2:**
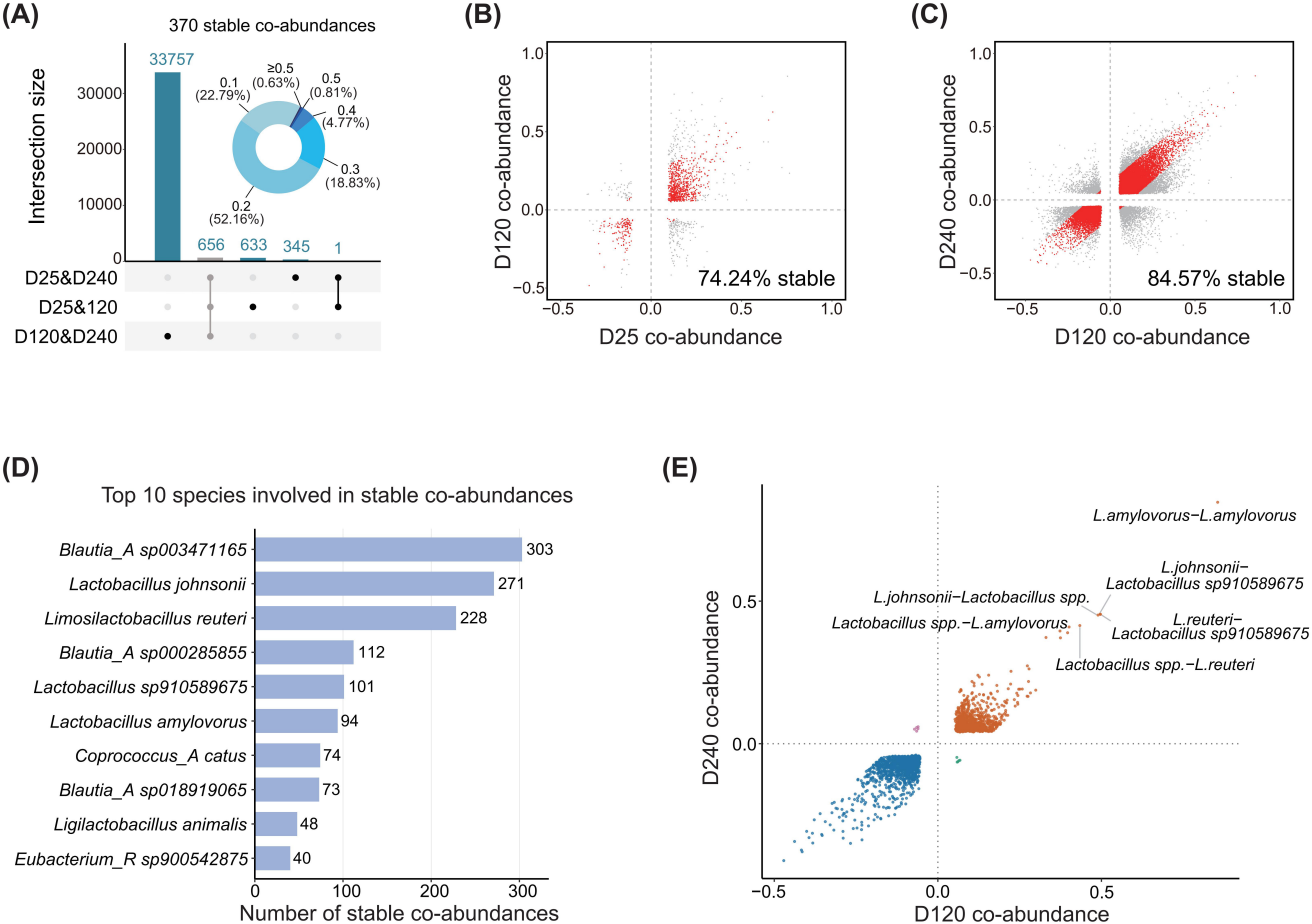
Stable co-abundance correlations across age stages in co-abundance networks involving beneficial commensal bacteria in the F6 population. **(A)** The number of co-abundance correlations shared among different age stages showing by an UpSet plot, and the distribution of co-abundance strength for 370 stably persisting co-abundance correlaions among the 656 co-abundance correlations detected across all three age stages, shown by a pie chart. The ring shows the number and proportion of co-abundance correlations at each range of correlation coefficients. **(B,C)** Scatter plots of correlation coefficients for co-abundance correlations shared between two age stages. **(B)** 1,289 co-abundance correlations shared between D25 and D120 stages, and **(C)** shows 34,413 co-abundance correlations shared between D120 and D240 stages. Red points represent conserved co-abundance correlations between the corresponding stages based on the Cochran-Q test with *P* > 0.05, and gray points represent non-conserved co-abundance correlations shared between two age stages with *P* < 0.05. **(D)** The top ten bacterial species showing the most stable co-abundance correlations among conserved correlations directly connected to beneficial commensal bacteria from D120 to D240. **(E)** Scatter plot of correlation coefficients for conserved co-abundance correlations directly connected to beneficial commensal bacteria from D120 to D240.

To assess the conservation of beneficial commensal bacteria involved co-abundance networks across three different age stages, we first identified 656 (0.32%, 656/206,008) co-abundance correlations that were present in all three age stages in the F6 population. Among these, 56.40% (370/656) showed no significant differences in correlation strength (correlation coefficient) among three age stages (Cochran-Q test, *P* > 0.05), suggesting that only a subset of these co-abundance correlations remained stable throughout the developmental period from pre-weaning to market in pigs (Figure 2A). These conserved co-abundance correlations were involved in 92 genera and were dominated by inter-genus interactions (Supplementary Figure S2A). Most co-abundance correlations were weak (74.95%, *r* < 0.2). Among these conserved co-abundance correlations, only 24 were directly linked to beneficial commensal bacteria. Co-abundance correlations associated with *Limosilactobacillus* and *Bifidobacterium* showed the highest degree of conservation (Supplementary Figure S2B), suggesting that only a limited number of beneficial commensal bacteria related interactions are stably maintained throughout pig development.

We further examined the conservation characteristics of co-abundance correlations in adult pigs (between D120 and D240). Compared with co-abundance patterns observed at all three age stages, the conservation of co-abundance correlations in adult pigs was substantially enhanced, with 84.57% (29,103/34,413) of co-abundance correlations remaining stable (Cochran-Q test, *P* > 0.05) (Figure 2C). These co-abundance correlations were also predominantly characterized by weak correlations, with 91.98% of correlation coefficients below 0.2. In adult pigs, 7.07% (2,058/29,103) of conserved co-abundance correlations were directly connected with beneficial commensal bacteria, comprising 49 species from 15 genera (Supplementary Table 3). Among these, *Blautia_A* and *Lactobacillus* were the two genera with the highest number of conserved co-abundance correlations. Within these conserved correlations, *L. reuteri* and *L. johnsonii* were core members of gut microbiota in adult pigs, and these two species also exhibited the greatest number of stable interactions (Figure 2D), suggesting that they might play key roles in maintaining microbial balance in the gut microecosystem of adult pigs. When we only focused on conserved co-abundance correlations among beneficial commensal bacterial species, distinct differences in correlation strength were observed, ranging from below 0.1 to above 0.4 (Figure 2E). Only a limited number of co-abundance correlations exhibited both stability and high correlation strength between two age stages, including co-abundance correlations between *L. reuteri* and *Lactobacillus sp910589675* or *Lactobacillus* spp., and between *L. johnsonii* and *Lactobacillus sp910589675* or *Lactobacillus* spp., all of which showed correlation coefficients exceeding 0.4 (Figure 2E), indicating highly stable and strong correlations among these lactic acid-producing species in adult pigs.

In contrast, the conservation of co-abundance correlations involving beneficial commensal bacteria was significantly lower in networks between 25 and 120 days, with 74.24% (957/1,289) of co-abundance correlations remaining stable between stages (Cochran-Q test, *P* > 0.05) (Figure 2B). Similarly, the strength of most of these stable co-abundance correlations was also weak (76.7% *r* < 0.2). Among these conserved co-abundance correlations, only 13.17% (126/957) were directly connected with beneficial commensal bacteria, which were involved in 23 species across 9 genera (Supplementary Table 4). *Lactobacillus* and *Limosilactobacillus* were the two genera containing the highest number of conserved co-abundance correlations (Supplementary Table 4). However, only 12 co-abundance correlations were composed exclusively of beneficial commensal bacteria, all of which exhibited positive coefficients below 0.25. This suggested that interactions between beneficial commensal bacteria that were stable across ages were generally weak during early development stages in pigs. In the F7 population, conservation patterns of co-abundance correlations across age stages were consistent with those observed in the F6 population (Supplementary Figure S3).

Overall, these results indicated that beneficial commensal bacteria involved co-abundance networks exhibited a form of dynamic stability across three different age stages. Across ages, large parts of co-abundance correlations among gut microbial species were conserved, but these correlations were generally weak and dominated by intergeneric interactions. This pattern suggested that weak interactions might play an important role in maintaining the long-term stability of the connection between beneficial commensal bacteria and other microbes in pig gut microecosystem communities. In contract, strong interactions are more likely to be age-specific.

### 3.3 Numerous age-shared correlations in beneficial commensal bacteria involved co-abundance networks were still variable across different developmental stages

Although many co-abundance correlations within beneficial commensal bacteria involved co-abundance networks were shared and conserved across three age stages, some shared correlations remained variable across ages. We therefore hypothesized that some shared co-abundance relationships might vary to respond to changes in host physiological states. To address this hypothesis, we performed a heterogeneity analysis of co-abundance correlations shared across age stages to evaluate their variation during different stages of host development.

**FIGURE 3:**
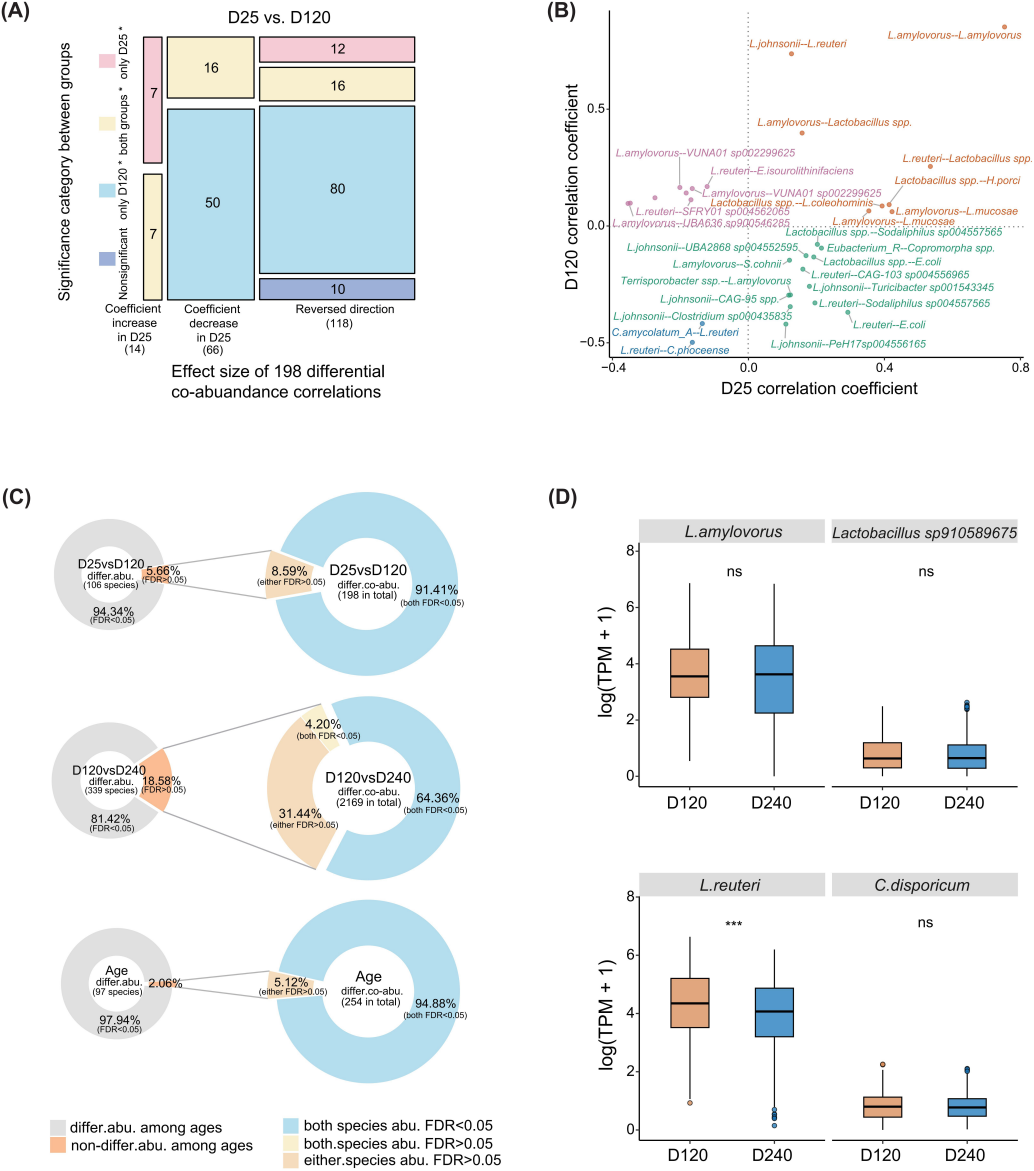
Variable co-abundance correlations across age stages in co-abundance networks involving beneficial commensal bacteria in the F6 population. **(A)** Summary of 198 differential co-abundance correlations between D25 and D120 stages identified using the Cochran-Q test with I^2^ > 75% and FDR < 0.05. The x-axis represents the effect size of differential co-abundance correlations between two age stages, and the y-axis categorizes co-abundance correlations based on their statistical significance at each age stage. Among 198 differential co-abundance correlations, 14 showed increased correlation strength at D25, including 7 that were significant only at D25 (FDR < 0.05). In contrast, 66 co-abundance correlations showed reduced correlation strength, of which 50 were not significant at D25 (FDR > 0.05). In addition, 118 co-abundance correlations exhibited a reversed correlation direction between stages. **(B)** Scatter plot of correlation coefficients for 31 variable co-abundance correlations directly connected to gut beneficial commensal bacteria between D25 and D120 stages. **(C)** Percentages of bacterial species showing differential and non-differential abundances between ages (the left panel) and their corresponding differential co-abundance correlations (the right panel). **(D)** Differences in species abundance involved in differential co-abundance correlations between D120 and D240 stages. *** FDR < 0.001, ns not significant. *P*-values were calculated using a two-sided Wilcoxon rank-sum test.

In the F6 population, 38.72% (254/656) of shared co-abundance correlations showed significant differences in co-abundance strength across three age stages (Cochran-Q test, I^2^ > 75% and FDR < 0.05), involving 61 genera (Supplementary Figure S4A). This result suggested that a substantial proportion of shared co-abundance correlations remained variable at different ages. Further comparison of differential characteristics of shared co-abundance correlations revealed variations not only in co-abundance strength but also in interaction direction. Compared to that at the age of 120 days, among 198 shared co-abundance correlations with differential characteristics identified between D25 and D120, 7.07% (14/198) showed the increase in co-abundance strength at D25, 33.33% (66/198) with decreased correlation strength, and 59.60% (118/198) were identified reversed interaction directions (Figure 3A). A comparable pattern was observed between D120 and D240 stages (Supplementary Figure S4B). These findings indicated that involving a subset of beneficial commensal bacteria in the pig gut remained dynamic even in adulthood.

Further analysis revealed variability in co-abundance correlation patterns both among beneficial commensal bacterial species and between beneficial commensal and potential pathogenic bacteria. Compared to D25, the strengths of multiple correlations significantly increased or decreased at D120, with 67.74% changing direction, indicating substantial network restructuring from the pre-weaning stage to adulthood (Figure 3B and Supplementary Table 5). From 120 to 240 days of age, beneficial commensal bacteria involved co-abundance networks continued to change (Supplementary Table 6). The strengths of co-abundance correlations among several beneficial commensal bacteria weakened from D120 to D240. In contrast, the co-abundance correlation between *B. pseudolongum* and *S. alactolyticus* was significantly enhanced from D120 to D240 and reached significance level only at 240 days (Supplementary Figure S4C). Another example, the correlation between *L. animalis* and the opportunistic pathogen *C. disporicum* shifted from negative correlation at D120 to a positive at D240 (Supplementary Figure S4C). These observations may provide insights into age-specific changes in interactions involving beneficial commensal bacteria in the healthy pig gut.

In the F7 population, similar patterns of differential characteristics of shared co-abundance correlations were observed. There were 68.89% (31/45) and 48.98% (360/735) of age-dependent co-abundance patterns in beneficial commensal bacteria involved co-abundance correlations shared between two age stages were reproducible in the F7 population between D25 and D120, and between D120 and D240 stages, respectively (Cochran-Q test, *P* > 0.05) (Supplementary Figure S4D). These results suggested that the patterns of co-abundance variation across age stages are partially reproducible in two independent populations.

To assess whether changes in microbial abundance drove differential co-abundance patterns across age stages, we compared the abundances of microbial species in co-abundance networks. We found that significant changes in the abundances of associated microbial species accompanied most differential co-abundance patterns (Figure 3C). However, a subset of differential co-abundance patterns was not directly driven by the changes in the abundances of associated microbial species. For example, across the D120 to D240 age range, microbial species with no significant differences in abundances accounted for 18.58% (63/339) of all species involved age-dependent differential co-abundance patterns, yet contributed to 35.68% (774/2,169) of differential co-abundance correlations. Among these, 27.02% (67/248) were directly related to beneficial commensal bacteria. For instance, *L. amylovorus* and *Lactobacillus sp910589675* did not differ significantly in abundance between D120 and D240 (Wilcoxon test, FDR > 0.05). However, their co-abundance patterns differed significantly between the two ages (Figure 3D). In addition, the potential pathogen *C. disporicum* was not differentially abundant between these two age stages, but its co-abundance correlation with *L. reuteri* differed significantly (Figure 3D). Similar results were observed at other age stages (Figure 3C). These findings suggested that although microbes showed no significant differences in abundance across age stages, their interaction patterns were substantially shifted.

### 3.4 Network topology identifies D120 as a critical window for beneficial commensal bacteria-mediated gut microbiota regulation

**FIGURE 4:**
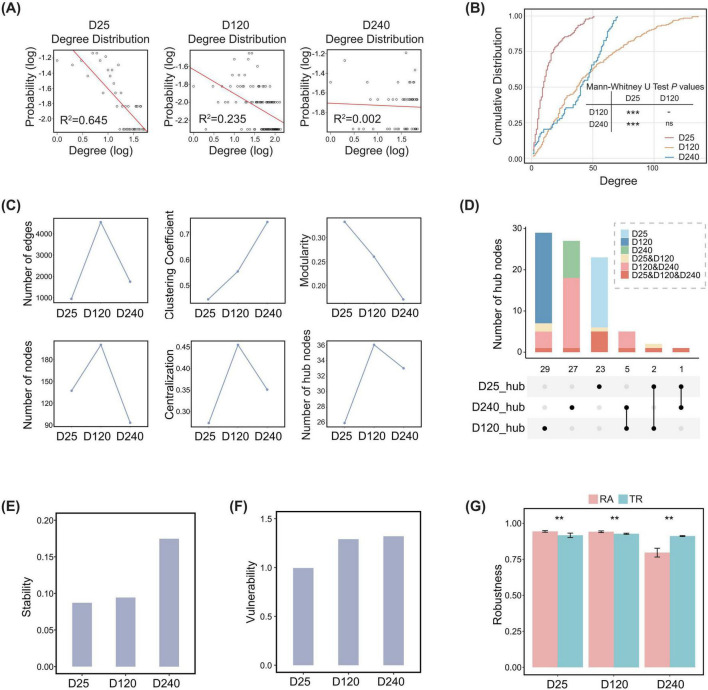
Topological properties of co-abundance networks involving beneficial gut commensal bacteria across three age stages. **(A)** Degree distributions of nodes in gut beneficial commensal bacteria involved co-abundance networks at D25, D120, and D240. The x-axis denotes the logarithm of node degree, and the y-axis denotes the logarithm of degree probability. The red line represents the fitted degree distribution. **(B)** Cumulative degree distribution curves of co-abundance networks involving beneficial gut commensal bacteria across three age stages. The x-axis represents node degree, and the y-axis represents the cumulative distribution. ***P < 0.001, ns not significant. **(C)** Dynamic changes in network topological metrics across three age stages, including the number of nodes, number of edges, clustering coefficient, modularity, centrality, and number of hub nodes in co-abundance networks involving beneficial gut commensal bacteria. **(D)** Intersections of hub nodes in beneficial commensal bacteria involved co-abundance networks across age stages and the number of these bacteria identified in multiple age stages. The upper bar plot shows the number of hub bacteria shared among networks at D25, D120, and D240. Stacked segments within each bar represent the number of species identified in multiple age stages in the corresponding intersections, including D25, D120, D240, D25 and D120, D120 and D240, and D25, D120 and D240. The lower dot-line plot illustrates the intersection relationships of hub nodes across age stages. **(E,F)** Comparisons of network stability **(E)** and vulnerability **(F)** of gut beneficial commensal bacteria involved co-abundance networks across three age stages. **(G)** Network robustness of gut beneficial commensal bacteria involved co-abundance networks across three age stages. Different evaluation strategies are indicated by different colors. RA: non-hub bacteria-based random attack, and TR: hub bacteria-based target removal. ** adjusted *P* < 0.01 after multiple-testing correction.

To identify age-related topological features of beneficial commensal bacteria involved co-abundance networks in the pig gut, microbial co-abundance correlations with correlation coefficients greater than 0.2 (robust correlations) were retained. The beneficial commensal bacteria involved co-abundance networks were then reconstructed for topological analysis. Based on node degree distributions, the beneficial commensal bacteria involved co-abundance networks at the D25 and D120 age stages followed power-law distributions, consistent with scale-free network characteristics. In contrast, the node degree distribution at the D240 stage deviated from a power-law distribution and more closely resembled that of a random network (Figure 4A). In addition, compared with that at the D25 stage, the degree distributions of beneficial commensal bacteria involved co-abundance networks at the D120 and D240 age stages showed distinct changes, indicating substantial alterations in the overall structure of gut probiotic involved communities before and after adulthood (Figure 4B). These results indicated that, from preweaning to adulthood, the co-abundance network of beneficial commensal bacteria in the pig gut gradually shifted from a scale-free to an approximately random structure. Notably, this structural transition appeared to occur around 120 days of age.

Further comparison of topological features of beneficial commensal bacteria involved co-abundance networks revealed that, compared with the that at the D25 and D240 age stages, the beneficial commensal bacteria involved co-abundance network at the D120 stage contained a greater number of nodes and edges, and a higher average degree, indicating a larger scale and a more complex structure at this stage (Figure 4C and Supplementary Figure S5A). It should likely be related to the higher bacterial diversity observed at the D120 stage (Supplementary Figure S5B). Across three age stages, the changes in the numbers of positive and negative interactions followed the same change trend in the total number of network edges. In addition, positive interactions outnumbered negative interactions at all three ages, indicating that the beneficial commensal bacteria involved co-abundance networks were predominantly composed of cooperative interspecies interactions (Supplementary Figure S5C).

With increasing pig age, the clustering coefficients of the beneficial commensal bacteria involved co-abundance networks gradually increased and reached a maximum at the D240 stage, whereas network modularity correspondingly decreased (Figure 4C). These changes indicated that, as pigs matured, communities of beneficial commensal bacteria became more tightly integrated, and overall network cooperation and integrity were enhanced. Meanwhile, network centralization and the number of hub bacteria peaked at the D120 stage and then declined markedly at the D240 stage, suggesting a reduced influence of hub bacteria from D120 to D240 (Figure 4C). Together, these results indicated that the beneficial commensal bacteria involved co-abundance networks in the pig gut underwent distinct changes from D120 to D240 stage.

Hub bacterial composition analysis further revealed age-specific characteristics of key nodes within the beneficial commensal bacteria involved co-abundance networks (Figure 4D and Supplementary Table 7). *L. johnsonii* was identified as a shared hub bacterium at the D120 and D240 stages and represented a highly connected node linking different network modules. *B. obeum* and *L. delbrueckii* were hub bacteria unique to the D25 stage. The network roles of most species varied across age stages. For example, *L. animalis* was commonly present in the gut of adult pigs. However, its role as beneficial commensal in co-abundance networks shifted with host age, changing from a hub bacterium at the D120 stage to a non-hub bacterium at the D240 stage (Supplementary Table 7). Similarly, *L. reuteri* was detected in fecal samples at all three age stages, but functioned as a hub bacterium only within the co-abundance networks at the D120 stage (Supplementary Table 7). Notably, beneficial commensal bacteria species were no longer identified in the hub bacteria unique to the D240 stage (Supplementary Table 7). These findings indicated that the key taxa co-abundance networks involving beneficial commensal bacteria differ across developmental stages, providing an important insight for future investigations into age-specific regulation of pig gut microbiome to improve economic traits.

We next evaluated the stability and vulnerability of beneficial commensal bacteria involved co-abundance networks across age stages. The values of both indices increased with age. Network stability was slightly higher at the D120 stage than at the D25 stage and reached its highest level at the D240 stage. Network vulnerability also peaked at the D240 stage, although it was only marginally higher than that observed at the D120 stage (Figures 4E,F). In addition, simulations of targeted removal of hub bacteria (TR) and random attack of non-hub bacteria (RA) showed that TR significantly reduced network robustness at the D25 and D120 stages (*P* < 0.05), whereas at the D240 stage, RA significantly reduced network robustness (*P* < 0.05) (Figure 4G). These results indicated that scale-free networks depend strongly on hub bacteria, whereas approximately random networks were more sensitive to random perturbations of non-hub bacteria.

In summary, topological changes in co-abundance networks involving beneficial commensal bacteria indicate that, among the three age stages examined, D120 represents a critical period for the structural and functional reorganization of the gut microbiota, whereas by D240 a new stable network state is established, in which beneficial commensal bacteria no longer dominate.

### 3.5 The gut microbial strains associated with average daily gain were organized in two competing guilds

**FIGURE 5:**
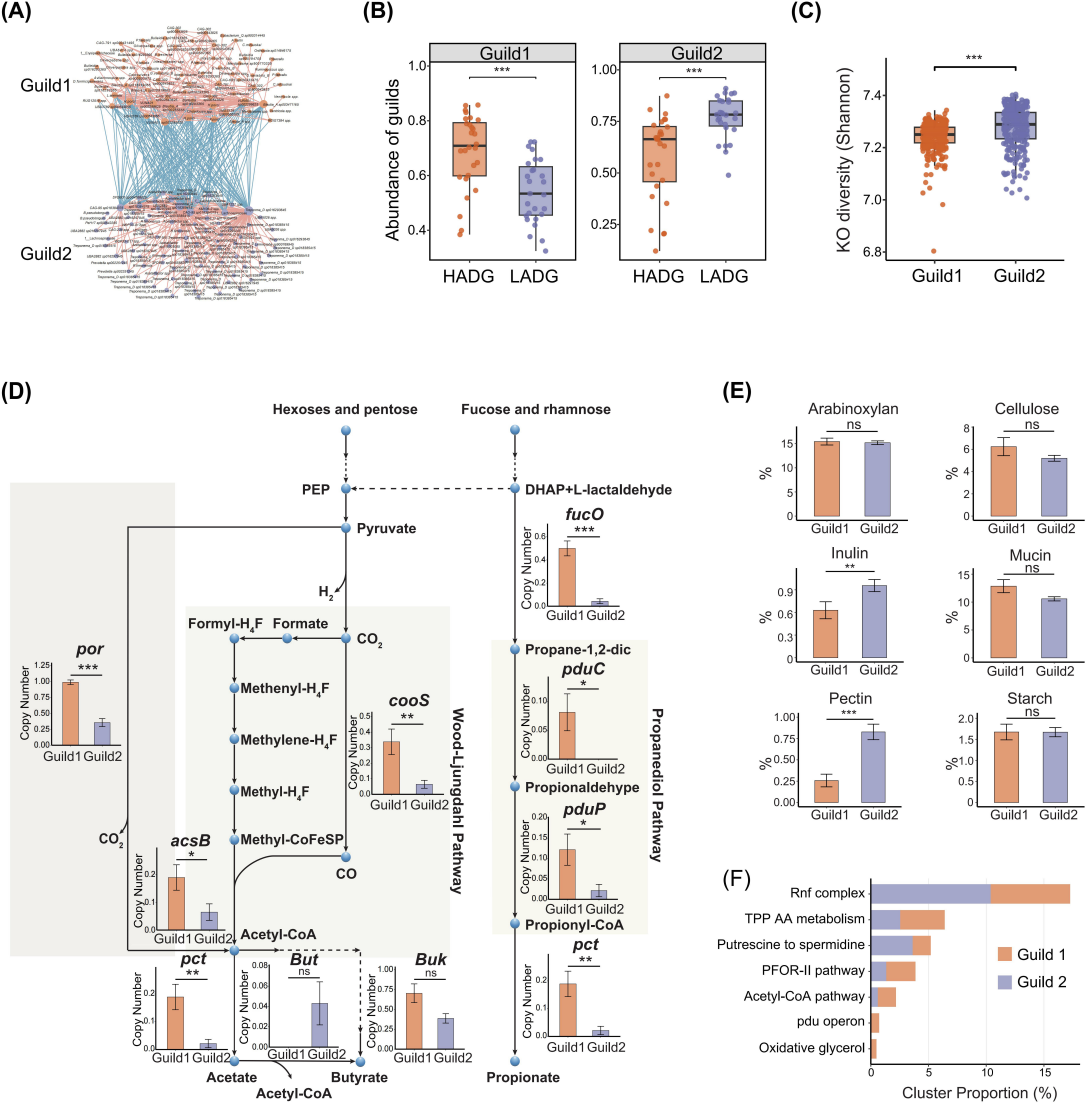
Gut microbial strains correlated with average daily gain (ADG) at the age of 120 days. **(A)** Co-abundance network of ADG-associated strains (HQMAGs) showing two competitive guilds. Co-abundance correlations among ADG-associated strains were calculated using FastSapr, and all significant associations with Benjamini-Hochberg adjusted *P* < 0.05 were retained. Edges represent correlations, with red and blue indicating positive and negative correlations, respectively. Node colors indicate Guild 1 (orange) and Guild 2 (purple). Guild 1 was contained of HQMAGs significantly and positively associated with ADG, whereas Guild 2 comprises HQMAGs significantly and negatively associated with ADG. **(B)** Differences in the abundances of two guilds in the pig gut with high ADG and low ADG, Guild 1 is shown in the left panel and Guild 2 in the right panel. ***P < 0.001. **(C)** Comparison of KEGG Ortholog (KO) diversity between two guilds assessed using Shannon diversity based on KOs detected in each genome. *P* values were calculated using a two-sided Wilcoxon rank-sum test. ***P < 0.001. **(D)** Differences between Guild 1 and Guild 2 in short-chain fatty acid (SCFA) production-related pathways and key gene copy numbers. Statistical differences were evaluated using a two-sided Mann-Whitney test. Acetate synthesis in these guilds occurs via two pathways, including the conversion of pyruvate to acetyl-CoA and the carbon monoxide branch of the Wood-Ljungdahl pathway, while propionate synthesis primarily relied on the propanediol metabolism pathway. *P < 0.05, **P < 0.01, ***P < 0.001, ns not significant. **(E)** Bar plots showing the proportions of CAZy genes associated with specific substrates, defined as the percentage of CAZy genes involved in the utilization of a given substrate relative to the total number of CAZy genes. **P < 0.01, ***P < 0.001, ns not significant. **(F)** Primary metabolic pathways detected with gutSMASH that differ significantly between Guild 1 and Guild 2.

At the D120 stage, we investigated the community structure of the gut microbiota associated with pig growth. Using the MaAsLin2 linear mixed-effects model, we identified 772 high-quality metagenome-assembled genomes (HQMAGs) that were significantly associated with average daily gain (ADG) from D120 to D240 in the F6 population. Among these HQMAGs, 445 showed significantly positive associations with ADG, while 327 showed significantly negative associations (FDR < 0.2). Considering the synergistic interaction among microbes with similar functions as a guild (Wu et al., 2024), we further performed a co-abundance analysis of these ADG-associated HQMAGs. We identified significant co-abundances among 167 HQMAGs (FDR < 0.05), which were organized into two guilds (Figure 5A and Supplementary Table 8). Within each guild, members showed exclusively significant positive correlations, whereas only negative correlations were detected between two guilds, suggesting a potentially competitive structural organization of ADG-associated bacteria within the pig gut ecosystem.

The Guild 1 was comprised of 74 HQMAGs that were significantly and positively associated with ADG (Supplementary Table 8). These 74 HQMAGs mainly included potentially beneficial bacteria, including short chain fatty acid-producing taxa, such as members in *Blautia_A*, *A. hallii*, and *L. animalis* (Supplementary Figure S6A). The Guild 2 contained 93 HQMAGs that were significantly and negatively associated with ADG (Supplementary Table 8). These negatively associated HQMAGs were dominated by members in *Treponema_D*, and also included *R. timonensis* and *B. pseudolongum* (Supplementary Figure S6A). In pigs with extreme ADG phenotypes, the abundance of Guild 1 was significantly higher than that of Guild 2 in high-ADG (HADG) pigs (*P* < 0.05), whereas low-ADG (LADG) pigs displayed a reverse pattern in the abundances of these two Guilds (*P* < 0.05) (Figure 5B). Furthermore, among individuals without significant differences in ADG, these two guilds had no significant difference in their abundance (*P* > 0.05) (Supplementary Figure S6B).

To explore the functional basis underlying the opposite associations of the two guilds with ADG, we compared their functional characteristics at the genomic level. Compared with the Guild 1, the Guild 2 exhibited greater functional diversity and higher richness in KEGG Orthologs (KOs) (Wilcoxon rank-sum test, *P* < 0.05) (Figure 5C and Supplementary Figure S6C), suggesting higher metabolic independence and enhanced adaptability in the context of niche competition and environmental fluctuations. Statistical analysis of KOs further showed that microbial genes in Guild 1 were significantly enriched in pathways related to translation, cell division, metabolic activity, and stress defense, including *por* and *efrA/B*, suggesting higher metabolic activity and a stronger capacity for homeostasis maintenance within this guild (Supplementary Figure S6D). In contrast, microbial genes in the Guild 2 were enriched in the pathways motility, membrane structure remodeling, environmental sensing, and stress regulation, including genes related to flagellar assembly and chemotaxis, such as *fliW*, *flgJ*, and *mcp*, as well as toxin antitoxin systems, reflecting enhanced environmental adaptability and niche competition (Supplementary Figure S6D).

Functional differentiation of gut microbiota between two guilds was particularly different in terms of carbohydrate utilization and short-chain fatty acid (SCFA) production capacity. Within Guild 1, key genes involved in acetate and propionate production were significantly enriched, including the pyruvate to acetyl-CoA pathway, the Wood-Ljungdahl pathway, and the propanediol metabolic pathway (Figure 5D). In contrast, microbial genes in the Guild 2 were enriched in CAZy genes for pectin and inulin utilization, while no significant differences were detected for genes involved in the utilization of several other complex polysaccharides (Figure 5E). Further analysis of primary metabolic gene clusters showed that microbial genes in the Guild 1 were mainly enriched in metabolic gene clusters related to acetate and propionate production, while genes in the Guild 2 were enriched in metabolic gene clusters of Rnf complex and the conversion of putrescine to spermidine. The accumulation of SCFAs in the gut might consequently inhibit members of Guild 2 by reducing intestinal local pH. The Rnf complex is primarily involved in energy conservation and redox balance in anaerobic bacteria (Tremblay et al., 2012), whereas polyamine metabolites such as putrescine are commonly associated with cellular stress responses and microbial autotrophic metabolism (Nakamura et al., 2018; Figure 5F). Putrescine supports cellular homeostasis and tissue development. Spermidine and spermine are indispensable for cell division, DNA synthesis and repair, and protein synthesis within a physiological concentration range. However, excessive accumulation, may pose health risks (Sagar et al., 2021).

In summary, microbes in the Guild 1 exhibited a greater capacity for acetate and propionate production, thereby enhancing host energy utilization. In contrast, the microbiota in the Guild 2 was associated with polyamine metabolism, with its metabolic products primarily supporting microbial energy maintenance. Differences in metabolic potential between microbes in two guilds might affect the efficiency of host energy acquisition by modulating carbon flow allocation within the gut microecosystem.

### 3.6 Genome-scale metabolic models reveal ADG-associated microbial metabolic interactions

**FIGURE 6:**
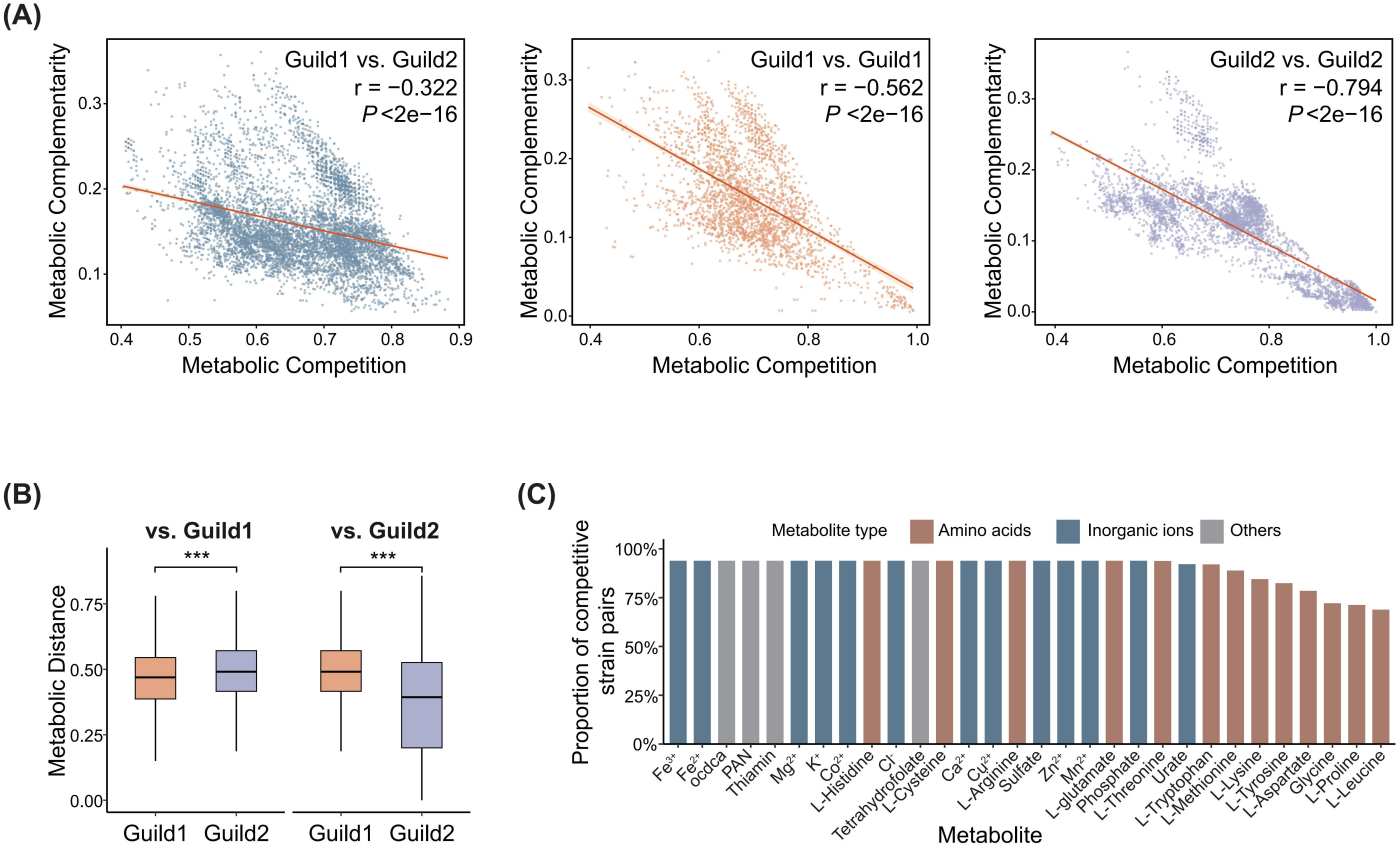
Metabolic interaction patterns among ADG-associated strains. **(A)** Metabolic competition and complementarity indices within Guild 1 and Guild 2, and between Guild 1 and Guild 2, calculated using PhyloMint. **(B)** Comparison of metabolic distances within and between Guild 1 and Guild 2. *P*-values were calculated using a two-sided Wilcoxon rank-sum test. ***P < 0.001. **(C)** The top 30 metabolites commonly required by metabolically competitive strain pairs between Guild 1 and Guild 2.

Previous studies have demonstrated that elucidating metabolic interactions among microorganisms can facilitate the modulation of microbial composition and their functional capacities, thereby influencing host physiology (Ma et al., 2025). Accordingly, we constructed GEMs encompassing all members of both guilds and applied a phylogenetically adjusted quantification approach to calculate the metabolic resource overlap (MRO; metabolic competition index) and the metabolic interaction potential (MIP; metabolic complementarity index) for all pairwise strain combinations (Lam et al., 2020). Our results indicated that metabolic competition and metabolic complementarity were significantly negatively correlated within and between guilds, suggesting that bacterial strains with greater overlap in metabolic resources tended to exhibit lower metabolic complementarity (Figure 6A). In contrast, the metabolic complementarity index was significantly and positively correlated with phylogenetic distance, suggesting that the more distant the strains were, the more likely to display metabolic dependence (Supplementary Figure S7A). This pattern was particularly obvious among strains within the Guild 2 (Supplementary Figure S7A). In addition, we identified several outlier strain pairs whose metabolic complementarity indices were markedly elevated relative to their phylogenetic distances, based on a Z-score threshold of 2.698, and these pairs were primarily observed among microbes from Guild 1 and Guild 2 (Supplementary Figure S7A).

Metabolic distances derived from the combined calculation of the metabolic competition index and the metabolic complementarity index further supported the above findings. Metabolic distances among strains within the same guild were significantly lower than those between guilds (Wilcoxon rank-sum test, *P* < 0.0001), and metabolic distances within Guild 2 were significantly lower than those within Guild 1 (Wilcoxon rank-sum test, *P* < 0.0001) (Figure 6B). These results indicated that members in the same guild shared similar niche preferences that favored the coexistence of functionally similar bacteria. This might be a major driver of ADG-associated microbial community assembly.

Our results indicated that ADG-associated strains between the two guilds predominantly exhibited a competitive metabolic profile at the community level. To identify the metabolites driving this competition, we identified metabolically competitive strain pairs between Guild 1 and Guild 2, accounting for 25% (1,721 of 6,882) of all strain pairs. Using flux balance analysis (FBA), we examined the overlapping metabolic demands for each metabolically competitive strain pair. These shared dependencies revealed potential competitive bottlenecks, with strain pairs from the two guilds primarily competing for amino acids and inorganic ions (Figure 6C).

MetaCyc pathway annotation showed that taxonomically closer strains shared more metabolic pathways, supporting the finding that these strains exhibited a higher degree of overlap in metabolic resource requirements (Supplementary Figure S7C). The proportion of core pathways, defined as those present in at least 90% of GEMs, was low (Guild 1: 1.46%; Guild 2: 4.80%). In contrast, the proportion of cloud pathways, present in only one GEM or in up to 10% of GEMs, was high (Guild 1: 46.34%; Guild 2: 51.01%). Together, these results suggested that both guilds exhibited high metabolic plasticity. Moreover, Guild 1 and Guild 2 shared some core metabolic pathways, including glycine biosynthesis III, oxalate degradation IV, queuosine biosynthesis II (queuine salvage), L-alanine biosynthesis III, and tRNA charging. These overlapped metabolic pathways suggested potential direct competition between the two guilds for these metabolic resources.

Comparison of uptake and secretion fluxes further revealed differences in metabolic resource allocation between the two guilds. Although no significant difference was observed in the numbers of predicted uptake and secreted metabolites between the two guilds (Wilcoxon rank-sum test, *P* > 0.05), clear divergence was observed in the utilization of specific metabolites (Supplementary Figure S7B). With respect to uptake fluxes, ribose and propionate were the most significantly enriched metabolites in Guild 1, whereas ornithine was the most significantly enriched metabolite in Guild 2. In terms of secretion, Guild 1 exhibited higher secretion of L-serine, while increased secretion of niacin and L-aspartate was observed in Guild 2 (Supplementary Figure S7D). Such differences in metabolic resource utilization and output might underlie the opposing associations between the two guilds and their distinct relationships with ADG. The result also provided insights into potential strategies to modulate ADG through targeted interventions on metabolite exchange within ADG-associated microbial communities.

## 4 Discussion

Previous studies have reported the positive effects of probiotics on host growth. However, there are significant differences in the performance of probiotics in different studies (Dowarah et al., 2017). Besides the differentiation of probiotic strains, colonization efficiency should be also another reason causing different performance of probiotics. Any microorganism including probiotic species exists in a gut microecosystem, and there are intricate interrelationships among microorganisms, which should influence the colonization and functional roles of probiotic species. To our knowledge, most of previous studies mainly focused on individual probiotic species (Xu et al., 2024), which provided limited insight into how probiotics established stable colonization and exerted functional capacities at the community level. Although gut microbiota heterogeneity across host developmental stages has been extensively described (de Goffau et al., 2022; Seo et al., 2023). Whether the colonization and their interaction with other microbes of probiotic species are specific to particular developmental stages have not been systematically elucidated. In this study, we analyzed 2,311 fecal metagenomic data collected from F6 and F7 pigs of a mosaic pig population across three developmental stages (D25, D120, and D240) spanning the developmental trajectory from pre-weaning to adulthood. Starting from the microbiota co-abundance networks, we systematically characterized the dynamic patterns of beneficial commensal bacteria within these networks across host ages and elucidated conserved and variable co-abundance features across developmental stages. Based on dynamic changes in network topology, we further identified the D120 stage as a potential critical period at which beneficial commensal bacteria contributed to the reorganization of gut network structure. We subsequently identified ADG-associated microbes at the strain level, and found that these microbes were organized into two competing guilds within the gut ecological network and exhibited significant differentiation in functional features and metabolic interaction patterns. The study provides important insights into age-related dynamic of beneficial commensal bacteria within co-abundance networks in the pig gut microbiome and offers a theoretical basis for the use of probiotics to regulate gut microbial composition.

We systematically investigated the conservation and dynamic changes of co-abundance networks involving beneficial commensal bacteria across ages. Several highly conserved beneficial commensal bacteria involved co-abundance correlations were found across three developmental stages. In particular, the co-abundance correlations among several lactic acid bacteria were highly stable and strong, suggesting that the simultaneous use of multiple lactic acid bacterial strains was conducive to colonization and to the regulation of pig gut microbiota. Although parts of beneficial commensal bacteria involved co-abundance networks were identified across three age stages, a substantial proportion of these shared co-abundance correlations remained variable, including the changes in correlation strength and direction, reflecting the significant effects of the changes in host physiological status, gut environments, and diets and metabolism following the ages on the interactions among bacterial species.

Studies across different hosts and gut locations have demonstrated that dynamic changes in the topology of gut microbial co-abundance networks reflected microbial community homeostasis (Wu et al., 2025) and provided a potential window into host physiological states and for regulating gut microbiota (Cheng et al., 2022; Li et al., 2023). Our results indicated that the beneficial commensal bacteria involved co-abundance networks at the D25 and D120 stages exhibited scale-free properties, with network robustness largely driven by a small number of highly connected hub bacteria. In contrast, at the D240 stage, network topology gradually shifted toward a near-random structure. The robustness of scale-free networks declined markedly following the removal of hub bacteria, whereas near-random networks were less affected. These findings were consistent with previous reports on the robustness of gut microbial co-abundance network structures and supported the differences in network types across developmental stages (Li et al., 2023). Notably, although the clustering coefficient of the beneficial commensal bacteria involved co-abundance network increased at the D240 stage, indicating a more tightly connected overall microbial community (Kajihara and Hynson, 2024), network centrality and the number of hub nodes were markedly reduced. This pattern suggested that network tightness was more likely attributable to a decentralization of connections rather than to strengthening interactions among beneficial commensal bacteria and its connected microbes. This topological change was consistent with host physiological characteristics, where pigs were in a period of relatively fast growth at the age of 120 days, whereas more fat deposition occurred at the age of 240 days. In particular, the topological reconfiguration observed from D120 to D240 suggested that microbial communities underwent adaptive shifts when pigs turned to the fat-accumulating phase from rapid growth. Beyond the redistribution of fat metabolism and nutrient absorption, this succession process might also have been driven by the changes in the gut physiological microenvironment during host development. Previous studies have suggested that gut motility serves as a link between host physiology and microbial ecosystems (Schwalbe et al., 2026). More specifically, gut motility actively shapes the intestinal environment by generating flow, shear, and localized nutrient gradients, thereby creating dynamic microniches (Schwalbe et al., 2026). From this perspective, gut motility might drive ecological filtering that affected microbial colonization, persistence, and interbacterial interactions (Wiles et al., 2016). Following the development of hosts, intestinal transit time may become longer and gut peristalsis may gradually slow down (Procházková et al., 2023). This process ultimately contributes to the transition in network structure. This possibility should be partly reflected in the taxonomic composition of the network. Members of Ruminococcaceae have been linked to prolonged intestinal transit time in previous studies (Procházková et al., 2023). In our data, *R. flavefaciens* was present at both stages, but emerged as a hub taxon only at D240, suggesting that it might have gained a stronger ecological advantage under conditions favoring prolonged substrate retention. In addition, microbial communities might also feedback on host physiology through metabolites and signaling molecules. Therefore, the observed network succession should reflect a dynamic host-microbiota feedback process in which host developmental changes remodel intestinal motility and microhabitats, while microbial metabolic activity further influences gut function and energy allocation. These findings provided a gut microecological basis for understanding the developmental stage specificity of beneficial commensal bacteria effects and highlighted the importance of considering host physiological status when designing probiotic-related interventions at different developmental stages.

Given a critical regulation window for gut microbiome at the age of 120 days, we therefore focused on the identification of key bacteria that might mediate the effects of the gut microbiota on ADG using the fecal metagenomic sequencing data at the age of 120 days. Using a guild-based analytical strategy, we constructed an ADG-associated co-abundance network with MAGs (Wu et al., 2024; Wu et al., 2021). Compared with the analyses conducted at the species or genus level, this approach offered higher resolution and allowed more precise characterization of ecological interactions among different guilds (Zhang and Zhao, 2016). HQMAGs significantly associated with ADG were organized into two competing guilds. This seesaw-like network organization characterized by two competing guilds has also been recognized as a core microbiome feature associated with ADG (Wu et al., 2024). This competitive relationship was supported by strain-level metabolic interaction patterns. Microbes in the Guild 1 were more related to metabolic activity and the maintenance of homeostasis. However, microbes in the Guild 2 showed enhanced capacity for environmental adaptation and regulation, and might be attributable to metabolic independence. Previous studies have shown that metabolic independence promoted the colonization and resilience of gut microbes under conditions of inflammation or nutritional stress (Watson et al., 2023). Furthermore, in the carbon flow allocation pattern analysis, microbial genes in Guild 1 were enriched in pathways for acetate and propionate biosynthesis, suggesting potential for SCFA production. Acetate has been shown to exert metabolic regulatory effects through activation of GPR43-mediated signaling pathways, including the induction of glucagon-like peptide 1 (GLP-1) and peptide YY (PYY) secretion in intestinal L-cells together with the regulation of insulin and glucose metabolism, as well as the inhibition of lipolysis and the promotion of energy storage in peripheral tissues (Robertson et al., 2005; Kimura et al., 2013). Propionate can serve as a substrate for intestinal gluconeogenesis and enhance host metabolic efficiency via signaling through the gut-brain neural axis (Koh et al., 2016). The enhanced capacity of Guild 1 for acetate and propionate synthesis might provide SCFAs as energy substrates and metabolic signaling molecules, thereby synergistically facilitating nutrient absorption and maintaining intestinal homeostasis, and ultimately promoting host growth. The accumulation of SCFAs in the gut might inhibit the members in the Guild 2 by lowering intestinal local pH, potentially forming competitive interactions between the two guilds. In contrast, microbial genes in the Guild 2 were enriched in the CAZymes associated with the degradation of pectin and inulin, indicating that it played an important role in the primary degradation of complex polysaccharides. However, microbes in both guilds should be able to decompose complex polysaccharides, leading to substrate-based competitive interactions. Therefore, differences in carbon source utilization strategies between the two guilds might lead to distinct patterns of carbon flow allocation and host energy acquisition efficiency, thereby giving rise to substrate-based competitive interactions within the gut ecosystem. This might contribute to their long-term stable coexistence within the same gut environment. This competitive relationship was supported by strain-level metabolic interaction patterns predicted by pFBA. Specifically, strains from Guild 1 and Guild 2 exhibited substantial overlap in metabolic demands, particularly for amino acids and inorganic ions, suggesting potential competition for shared nutrient resources. This observation was consistent with previous *in vivo* metabolomics studies, which have demonstrated that nutrient availability can reshape microbial competition, particularly among microorganisms with overlapping metabolic capabilities (Zhao et al., 2023; Zhao et al., 2025b). In addition, the gas molecules produced by microbial fermentation also contribute to the competitive dynamics between the two guilds (Zhao et al., 2025a,Qi et al., 2024). Guild 2 was dominated by *Treponema* spp., members of which have been reported to be associated with potential H2S production (Wu et al., 2026). In contrast, Guild 1 was enriched in SCFA producers. The accumulation of SCFAs might acidify the gut environment and exert antimicrobial effects, thereby potentially constraining microbial groups associated with the production of H2S, indole, and endotoxin (Zhao et al., 2024). Therefore, modulation of gaseous metabolites and local physicochemical conditions should represent an additional ecological layer that influences community stability and substrate utilization preferences, thereby reinforcing competitive interactions between the two guilds. The GEMs and pFBA analysis used in this study were based on genome-derived metabolic reconstructions, which primarily reflected the potential metabolic capabilities of microbial strains under *in vitro* conditions. Although such models provide valuable system-level insights, they could not fully capture the complexity of microbial interactions within the host guts (Quinn-Bohmann et al., 2025). Thus, integrating GEM-based predictions with experimental validation is essential. Future studies incorporating *in vivo* metabolomics and flux-based approaches will be valuable for further validating and refining these findings. However, our study offerred a basis for generating testable hypotheses. Together, these findings provided new insights into the complex relationships between the gut microbiome and host ADG.

In summary, we characterized the dynamics of co-abundance networks involving beneficial commensal bacteria in the pig gut microbiota across host ages and elucidated their conserved and variable features across developmental stages. Age-shared co-abundance correlations among beneficial commensal bacteria in co-abundance networks remained variable across developmental stages, including changes in correlation strength and interaction direction. Topological changes in co-abundance networks involving beneficial commensal bacteria indicated that D120 represents a critical window for the regulation of the pig gut microbiota among the three age stages examined. Furthermore, we identified 772 high-quality MAGs that were significantly associated with ADG from D120 to D240 and formed two negatively correlated guilds. We also inferred the ecological interaction mechanisms of ADG-associated microbial communities. This study had several limitations that needed to be addressed in future study. First, the ADG-associated guilds identified in this study would require to be validated in multiple pig populations with comparable growth phenotypes. Second, individual feed intake was not measured in this study. Nevertheless, all animals were maintained under the same housing and management conditions and were fed the same diet within each age group, which might minimize the effect of variables related to feeding management on the gut microbiota of experimental pigs. Third, interactions between beneficial commensal bacteria and other bacterial species in the pig gut were just inferred from co-abundance correlations. Further *in vitro* or *in vivo* experiments should be performed to confirm the interactions. However, the findings from this study provided a scientific basis for future probiotic interventions in the gut microbiome to improve pig growth in pig production and promoted the development of customized probiotic consortia tailored to different growth phases.

## Data Availability

Metagenome-assembled genomes (MAGs) have been deposited and are publicly available in the National Genomics Data Center under Genome Sequence Archive with bioProject accession number PRJCA058837 (http://ngdc.cncb.ac.cn/bioproject/browse/PRJCA058837). Metagenomic sequencing data were publicly available in the China National GeneBank DataBase (CNGBdb) under accession code CNP0000824 (https://db.cngb.org/data_resources/project/CNP0000824).

## Supplementary material

Supplementary Figure S1 Characteristics of co-abundance networks involving beneficial commensal bacteria in the gut microbiota across three age stages in the F7 population. **(A)** Module similarity of gut microbial co-abundance networks between pairwise age stages. The left panel shows boxplots of similarity coefficients derived from pairwise comparisons of modules between pairwise age stages, and the right panel shows a heatmap of mean module similarity across the three age stages. **(B)** Principal coordinates analysis (PCoA) based on Bray-Curtis distances illustrating differences in gut microbial community composition at D25, D120, and D240. PERMANOVA was performed using 999 permutations, with Benjamini-Hochberg adjusted *P* < 0.05. **(C,D)** Numbers of shared and specific co-abundance correlations and the corresponding species in co-abundance networks involving beneficial commensal bacteria at D25, D120, and D240, including the number of species involved in co-abundance correlations **(C)** and the number of co-abundance correlations identified **(D)**. **(E,F)** Changes in co-abundance patterns with increasing correlation coefficients in the F7 population. **(E)** The number of species involved in co-abundance correlations, and **(F)** the number of co-abundance correlations shared among different age-stages following the correlation coefficients. The x-axis represents the co-abundance correlation coefficients. **(G)** Inter-module similarity between the whole gut bacterial co-abundance network and the subnetwork involving beneficial commensal bacteria in the F7 population. Bubble plots illustrate the similarity between modules of the beneficial commensal bacteria involved co-abundance subnetwork and modules of the whole bacterial co-abundance network at D25, D120, and D240. Bubble size indicates the values of inter-module similarity, and color represents the age stage.

Supplementary Figure S2 Genus-level co-abundance correlations conserved across three age stages and interactions associated with beneficial commensal bacteria in the F6 population. **(A)** Genus-level interaction patterns of 370 co-abundance correlations conserved across three age stages in the F6 population. **(B)** Twenty-four of 370 conserved co-abundance correlations were directly linked to beneficial commensal bacteria involved in genera across three age stages. Each dot represents a microbial genus. Each line represents conserved co-abundance correlations either within the same genus or between different genera. Line width and color intensity indicate the number of significant correlations between two genera.

Supplementary Figure S3 Stable co-abundance correlations across age stages in co-abundance networks involving beneficial commensal bacteria in the F7 population. **(A)** UpSet plot showing the number of co-abundance correlations shared among different age stages, and a pie chart showing the distribution of co-abundance strength for 626 stably persisting co-abundance correlations out of 1464 co-abundance correlations detected at three age stages. The ring shows the number and proportion of co-abundance correlations at each range of correlation coefficients. **(B)** Genus-level interaction patterns of 626 co-abundance correlations conserved across the three age stages in the F7 population. **(C)** Thirty-seven of the 626 conserved co-abundance correlations were directly linked to genera involving beneficial commensal bacteria across the three age stages. Each dot represents a microbial genus. Each line represents conserved co-abundance correlations either within the same genus or between different genera. Line width and color intensity indicate the number of such correlations. **(D,E)** Scatter plots of correlation coefficients for co-abundance correlations shared between two age stages. **(D)**,472 co-abundance correlations shared between D25 and D120 stages, and **(E)** 45,122 co-abundance correlations shared between D120 and D240 stages. Red points represent conserved co-abundance correlations between the corresponding stages based on the Cochran-Q test with *P* > 0.05, and gray points represent non-conserved co-abundance correlations shared between two age stages with *P* < 0.05.

Supplementary Figure S4 Variable co-abundance correlations across age stages in co-abundance networks involving beneficial commensal bacteria. **(A)** Proportions of significantly differential and stable co-abundance correlations among 656 correlations shared across three age stages in the F6 population. **(B)** Summary of 2,169 differential co-abundance correlations between D120 and D240 stages identified using the Cochran-Q test with I^2^ > 75% and FDR < 0.05. The X-axis represents the effect size of differential co-abundance correlations between the two age stages, and the Y-axis categorizes co-abundance correlations based on their statistical significance at each age stage. **(C)** Scatter plot of correlation coefficients for 248 variable co-abundance correlations directly connected to gut beneficial commensal bacteria between D120 and D240 stages. **(D)** Replication of differential co-abundance correlations between F6 and F7 populations. The left panel shows replication of differential co-abundance correlations identified between the D25 and D120 stages in the F6 population in the corresponding stages of the F7 population, whereas the right panel shows replication of differential co-abundance correlations identified between the D120 and D240 stages in the F6 population in the corresponding stages of the F7 population. The x-axis represents correlation coefficients of differential co-abundance correlations in the F6 population, and the y-axis represents correlation coefficients of the corresponding co-abundance correlations in the F7 population. Each point represents a single differential co-abundance correlation. Red points indicate reproducible co-abundance correlations according to the Cochran-Q test (*P* > 0.05), whereas gray points indicate non-reproducible co-abundance correlations (*P* < 0.05).

Supplementary Figure S5 Topological properties of co-abundance networks involving beneficial commensal bacteria in the gut microbiota across three age stages. **(A)** Dynamic changes in the average node degree across three age stages. **(B)** Alpha diversity of gut microbial communities across three age stages. **(C)** Positive and negative interactions in gut beneficial commensal bacteria involved co-abundance networks across three age stages.

Supplementary Figure S6 Gut microbial strains correlated with average daily gain (ADG) at the age of 120 days. **(A)** Phylogenetic tree of the 167 ADG-associated strains (HQMAG). Branches are colored according to the family-level taxonomy of each HQMAG. Outer red and blue labels indicate HQMAGs identified as positively or negatively associated with ADG, respectively. Black bars surrounding the circular tree indicate the genus-level taxonomic information. **(B)** Differences in the abundances of two guilds in the pig gut without significant differences in ADG, Guild 1 is shown in the left panel and Guild 2 in the right panel. ns not significant. **(C)** Comparison of KEGG Orthologs (KOs) counts between two guilds based on annotations in individual genomes. *P* values were calculated using a two-sided Wilcoxon rank-sum test. *** *P* < 0.001. **(D)** Heatmap showing the distribution of the top 20 KOs significantly associated with Guild 1 or Guild 2. Columns represent ADG-associated strains colored by guild (Guild 1 or Guild 2). KOs are clustered by complete-linkage hierarchical clustering based on presence-absence patterns.

Supplementary Figure S7 Comparison of metabolic indices, metabolic potential, metabolic fluxes, and metabolite exchange between two guilds. **(A)** The correlations between metabolic complementarity index and phylogenetic distance within Guild 1 and Guild 2, and between Guild 1 and Guild 2. Significant complementary outliers are highlighted in red based on a Z-score threshold of 2.698. **(B)** Differences between Guild 1 and Guild 2 in the number of metabolites predicted from uptake fluxes (left panel) or secretion fluxes (right panel). *P*-values were calculated using a two-sided Wilcoxon rank-sum test. ns not significant. **(C)** Heatmap of metabolic pathway presence-absence across subsystems in HQMAGs from both guilds. **(D)** Presence-absence patterns of metabolites significantly associated with Guild 1 or Guild 2 in each ADG-associated strain. Columns represent strains colored by guild. Metabolites are clustered by complete-linkage hierarchical clustering based on presence-absence patterns and colored by metabolic flux type, including uptake and secretion.

Supplementary Table 1 Dietary components of commercial formula diets for pigs at different ages.

Supplementary Table 2 Beneficial commensal bacteria used in the co-abundance network analysis in this study.

Supplementary Table 3 Conserved co-abundance correlations directly connected to species involving beneficial commensal bacteria from the age of D120 to D240.

Supplementary Table 4 Conserved co-abundance correlations directly connected to species involving beneficial commensal bacteria from the age of D25 to D120.

Supplementary Table 5 Variable co-abundance correlations directly linked to species involving beneficial commensal bacteria from the age of D25 to D120.

Supplementary Table 6 Variable co-abundance correlations directly linked to species involving beneficial commensal bacteria from the age of D120 to D240.

Supplementary Table 7 Intersection of hub nodes and their prevalence across age stages in co-abundance networks involving beneficial commensal bacteria.

Supplementary Table 8 Two competing guilds of average daily gain-associated microbes (MAGs).
