# Supplementary figures and images for: Topological characteristics and longitudinal dynamics of co-abundance networks involving beneficial commensal bacteria in the pig gut microbiome and its association with average daily gain
Source: Front Microbiol. 2026 Apr 29;17:1818141. doi: 10.3389/fmicb.2026.1818141 (PMC13168023; doi:10.3389/fmicb.2026.1818141)

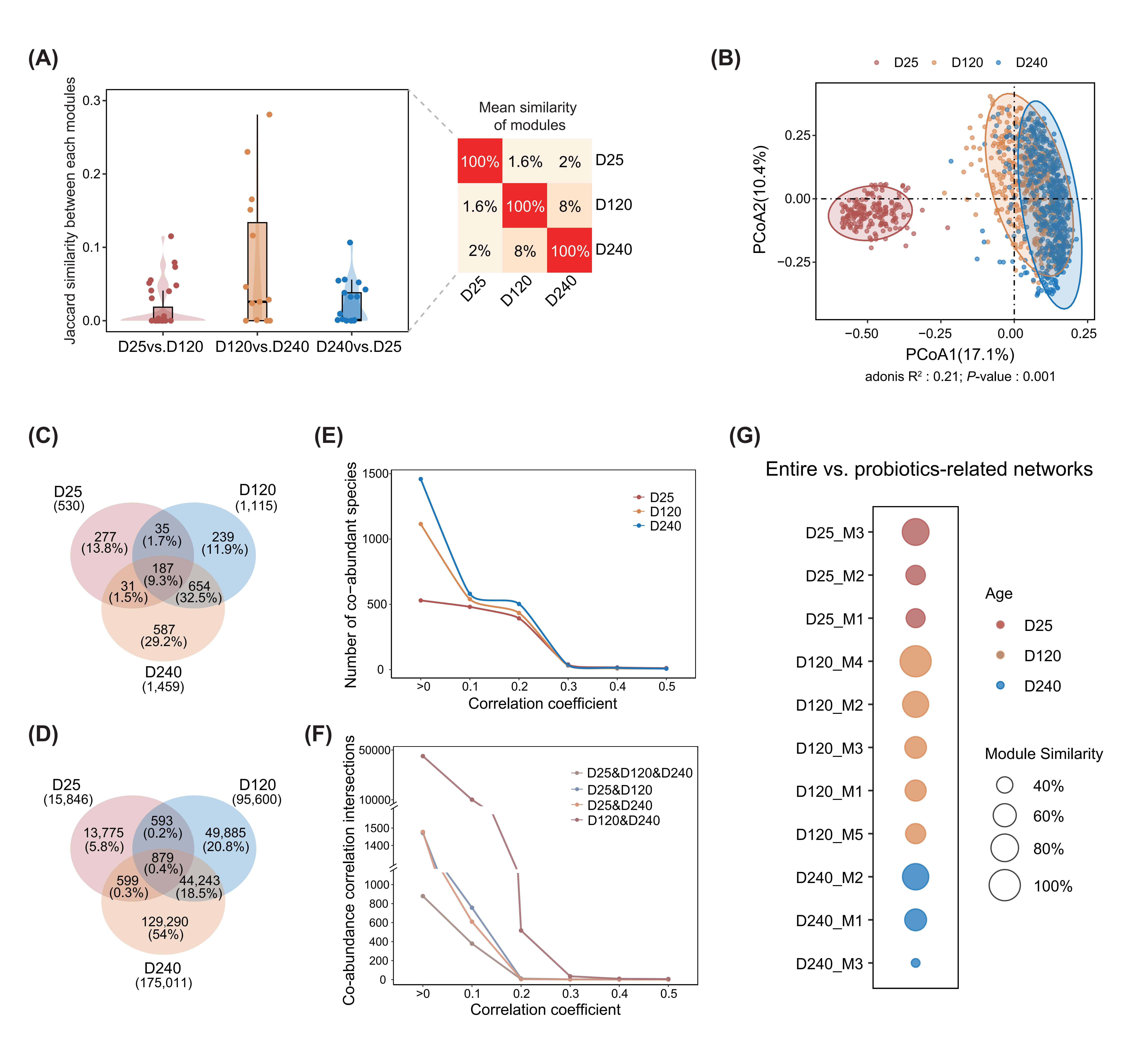

Supplement: Supplementary Figure S1 — Characteristics of co-abundance networks involving beneficial commensal bacteria in the gut microbiota across three age stages in the F7 population. (A) Module similarity of gut microbial co-abundance networks between pairwise age stages. The left panel shows boxplots of similarity coefficients derived from pairwise comparisons of modules between pairwise age stages, and the right panel shows a heatmap of mean module similarity across the three age stages. (B) Principal coordinates analysis (PCoA) based on Bray-Curtis distances illustrating differences in gut microbial community composition at D25, D120, and D240. PERMANOVA was performed using 999 permutations, with Benjamini-Hochberg adjusted P < 0.05. (C,D) Numbers of shared and specific co-abundance correlations and the corresponding species in co-abundance networks involving beneficial commensal bacteria at D25, D120, and D240, including the number of species involved in co-abundance correlations (C) and the number of co-abundance correlations identified (D). (E,F) Changes in co-abundance patterns with increasing correlation coefficients in the F7 population. (E) The number of species involved in co-abundance correlations, and (F) the number of co-abundance correlations shared among different age-stages following the correlation coefficients. The x-axis represents the co-abundance correlation coefficients. (G) Inter-module similarity between the whole gut bacterial co-abundance network and the subnetwork involving beneficial commensal bacteria in the F7 population. Bubble plots illustrate the similarity between modules of the beneficial commensal bacteria involved co-abundance subnetwork and modules of the whole bacterial co-abundance network at D25, D120, and D240. Bubble size indicates the values of inter-module similarity, and color represents the age stage. [file Image_1.jpeg]

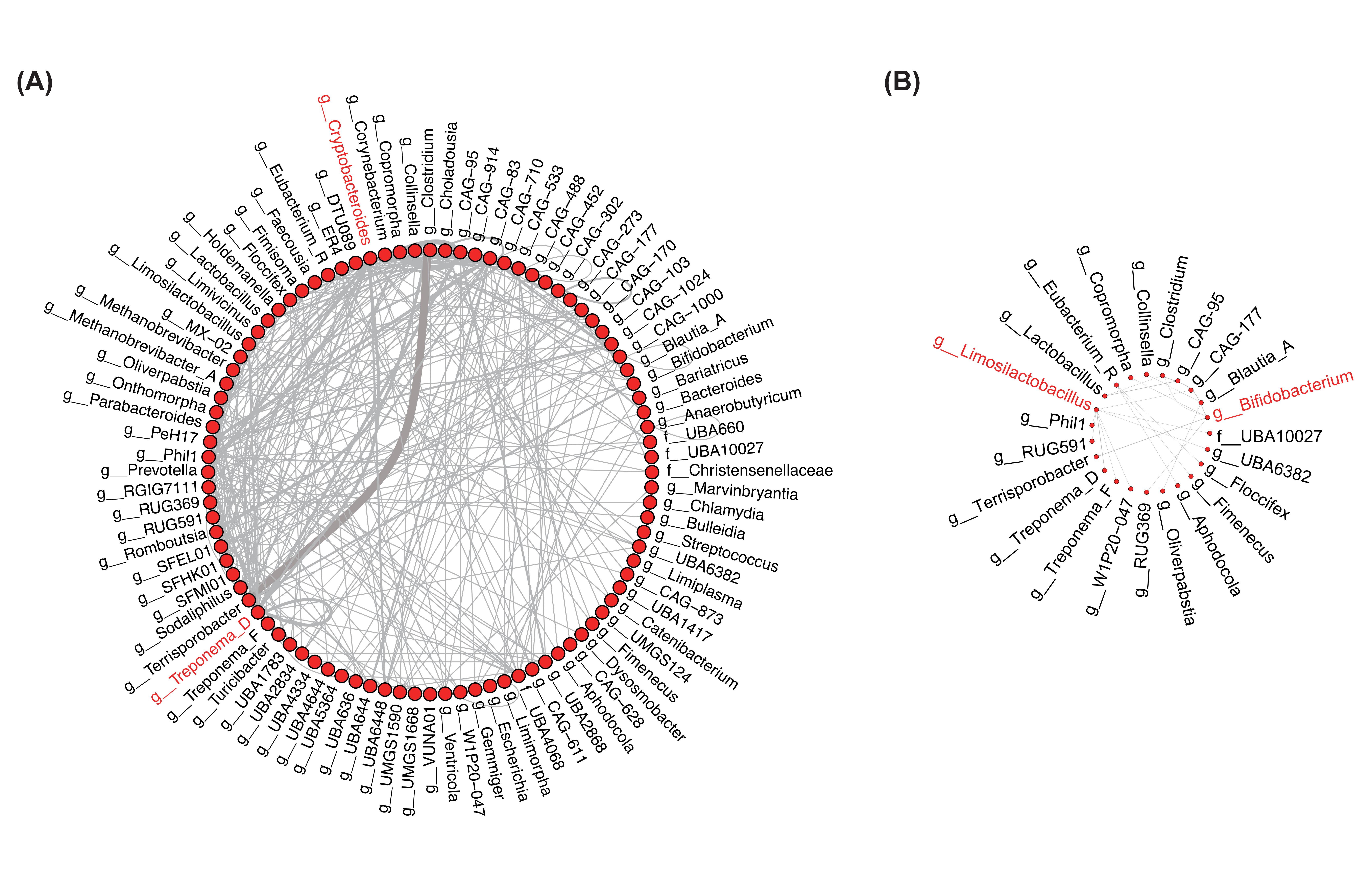

Supplement: Supplementary Figure S2 — Genus-level co-abundance correlations conserved across three age stages and interactions associated with beneficial commensal bacteria in the F6 population. (A) Genus-level interaction patterns of 370 co-abundance correlations conserved across three age stages in the F6 population. (B) Twenty-four of 370 conserved co-abundance correlations were directly linked to beneficial commensal bacteria involved in genera across three age stages. Each dot represents a microbial genus. Each line represents conserved co-abundance correlations either within the same genus or between different genera. Line width and color intensity indicate the number of significant correlations between two genera. [file Image_2.jpeg]

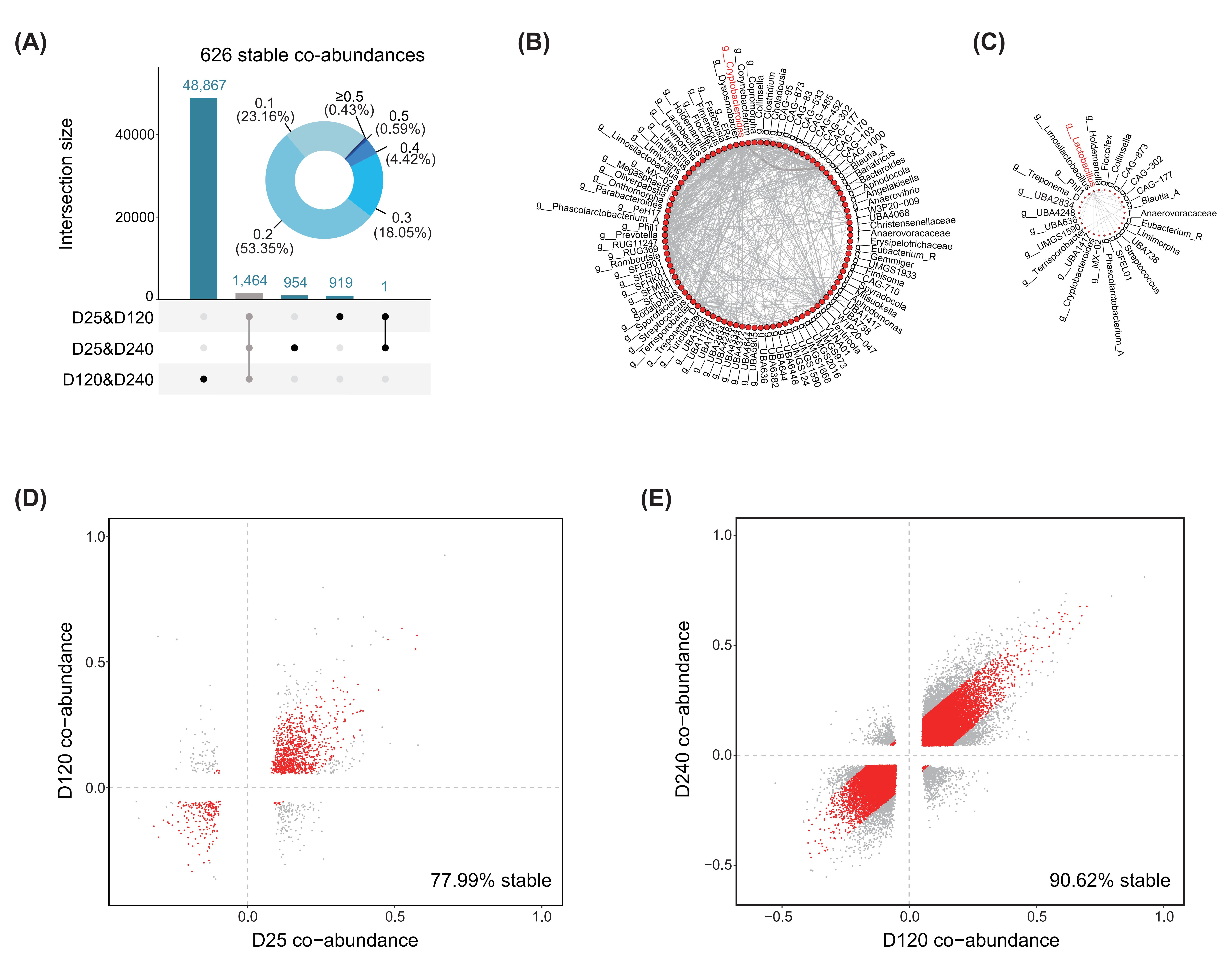

Supplement: Supplementary Figure S3 — Stable co-abundance correlations across age stages in co-abundance networks involving beneficial commensal bacteria in the F7 population. (A) UpSet plot showing the number of co-abundance correlations shared among different age stages, and a pie chart showing the distribution of co-abundance strength for 626 stably persisting co-abundance correlations out of 1464 co-abundance correlations detected at three age stages. The ring shows the number and proportion of co-abundance correlations at each range of correlation coefficients. (B) Genus-level interaction patterns of 626 co-abundance correlations conserved across the three age stages in the F7 population. (C) Thirty-seven of the 626 conserved co-abundance correlations were directly linked to genera involving beneficial commensal bacteria across the three age stages. Each dot represents a microbial genus. Each line represents conserved co-abundance correlations either within the same genus or between different genera. Line width and color intensity indicate the number of such correlations. (D,E) Scatter plots of correlation coefficients for co-abundance correlations shared between two age stages. (D),472 co-abundance correlations shared between D25 and D120 stages, and (E) 45,122 co-abundance correlations shared between D120 and D240 stages. Red points represent conserved co-abundance correlations between the corresponding stages based on the Cochran-Q test with P > 0.05, and gray points represent non-conserved co-abundance correlations shared between two age stages with P < 0.05. [file Image_3.jpeg]

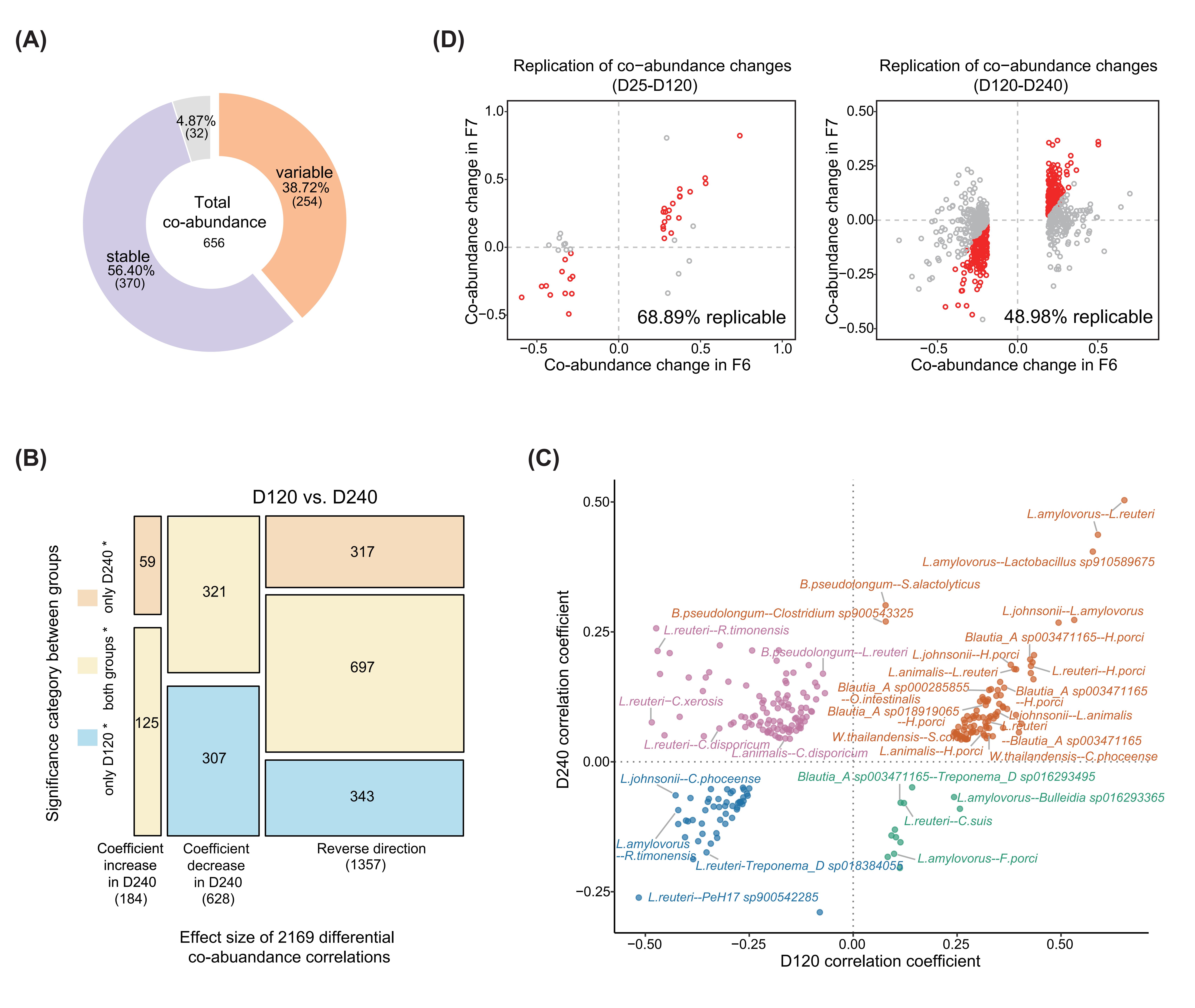

Supplement: Supplementary Figure S4 — Variable co-abundance correlations across age stages in co-abundance networks involving beneficial commensal bacteria. (A) Proportions of significantly differential and stable co-abundance correlations among 656 correlations shared across three age stages in the F6 population. (B) Summary of 2,169 differential co-abundance correlations between D120 and D240 stages identified using the Cochran-Q test with I2 > 75% and FDR < 0.05. The X-axis represents the effect size of differential co-abundance correlations between the two age stages, and the Y-axis categorizes co-abundance correlations based on their statistical significance at each age stage. (C) Scatter plot of correlation coefficients for 248 variable co-abundance correlations directly connected to gut beneficial commensal bacteria between D120 and D240 stages. (D) Replication of differential co-abundance correlations between F6 and F7 populations. The left panel shows replication of differential co-abundance correlations identified between the D25 and D120 stages in the F6 population in the corresponding stages of the F7 population, whereas the right panel shows replication of differential co-abundance correlations identified between the D120 and D240 stages in the F6 population in the corresponding stages of the F7 population. The x-axis represents correlation coefficients of differential co-abundance correlations in the F6 population, and the y-axis represents correlation coefficients of the corresponding co-abundance correlations in the F7 population. Each point represents a single differential co-abundance correlation. Red points indicate reproducible co-abundance correlations according to the Cochran-Q test (P > 0.05), whereas gray points indicate non-reproducible co-abundance correlations (P < 0.05). [file Image_4.jpeg]

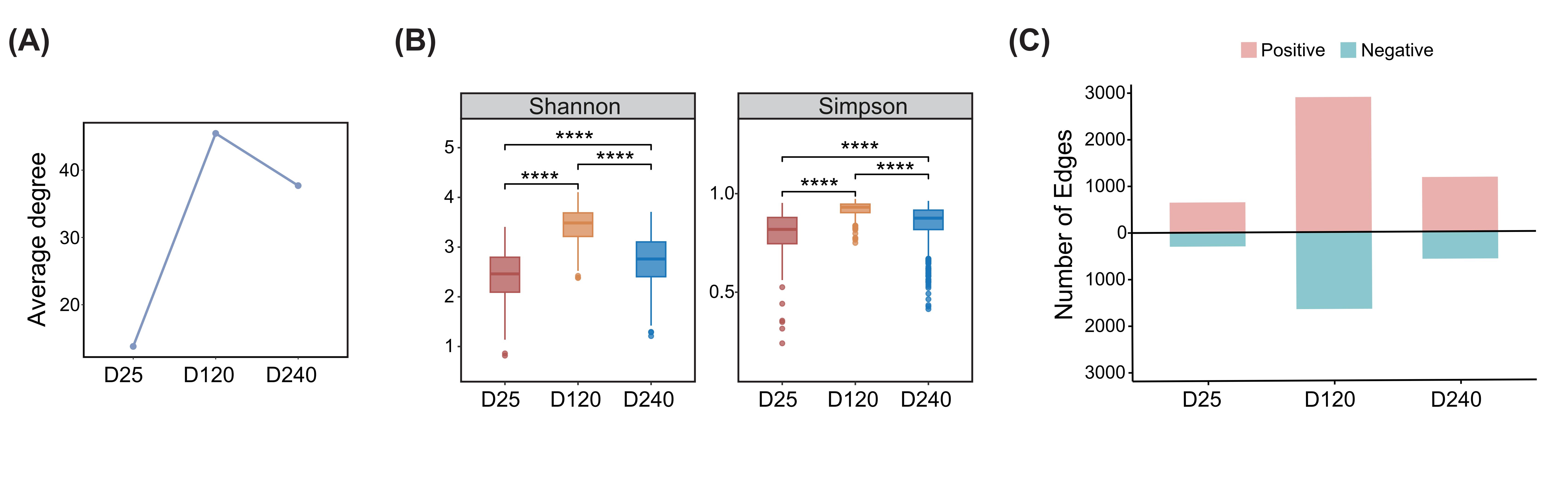

Supplement: Supplementary Figure S5 — Topological properties of co-abundance networks involving beneficial commensal bacteria in the gut microbiota across three age stages. (A) Dynamic changes in the average node degree across three age stages. (B) Alpha diversity of gut microbial communities across three age stages. (C) Positive and negative interactions in gut beneficial commensal bacteria involved co-abundance networks across three age stages. [file Image_5.jpeg]

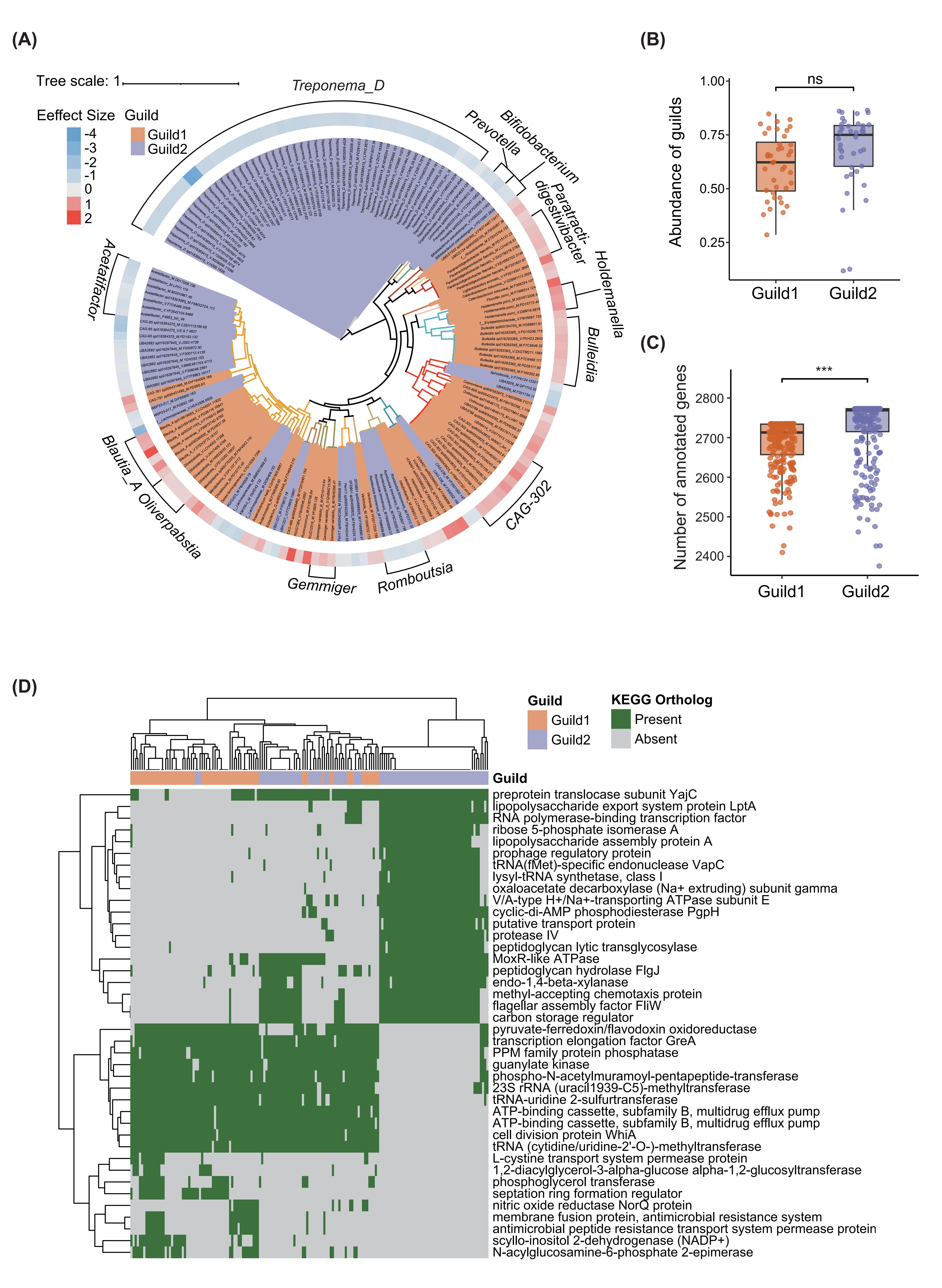

Supplement: Supplementary Figure S6 — Gut microbial strains correlated with average daily gain (ADG) at the age of 120 days. (A) Phylogenetic tree of the 167 ADG-associated strains (HQMAG). Branches are colored according to the family-level taxonomy of each HQMAG. Outer red and blue labels indicate HQMAGs identified as positively or negatively associated with ADG, respectively. Black bars surrounding the circular tree indicate the genus-level taxonomic information. (B) Differences in the abundances of two guilds in the pig gut without significant differences in ADG, Guild 1 is shown in the left panel and Guild 2 in the right panel. ns not significant. (C) Comparison of KEGG Orthologs (KOs) counts between two guilds based on annotations in individual genomes. P values were calculated using a two-sided Wilcoxon rank-sum test. *** P < 0.001. (D) Heatmap showing the distribution of the top 20 KOs significantly associated with Guild 1 or Guild 2. Columns represent ADG-associated strains colored by guild (Guild 1 or Guild 2). KOs are clustered by complete-linkage hierarchical clustering based on presence-absence patterns. [file Image_6.jpeg]

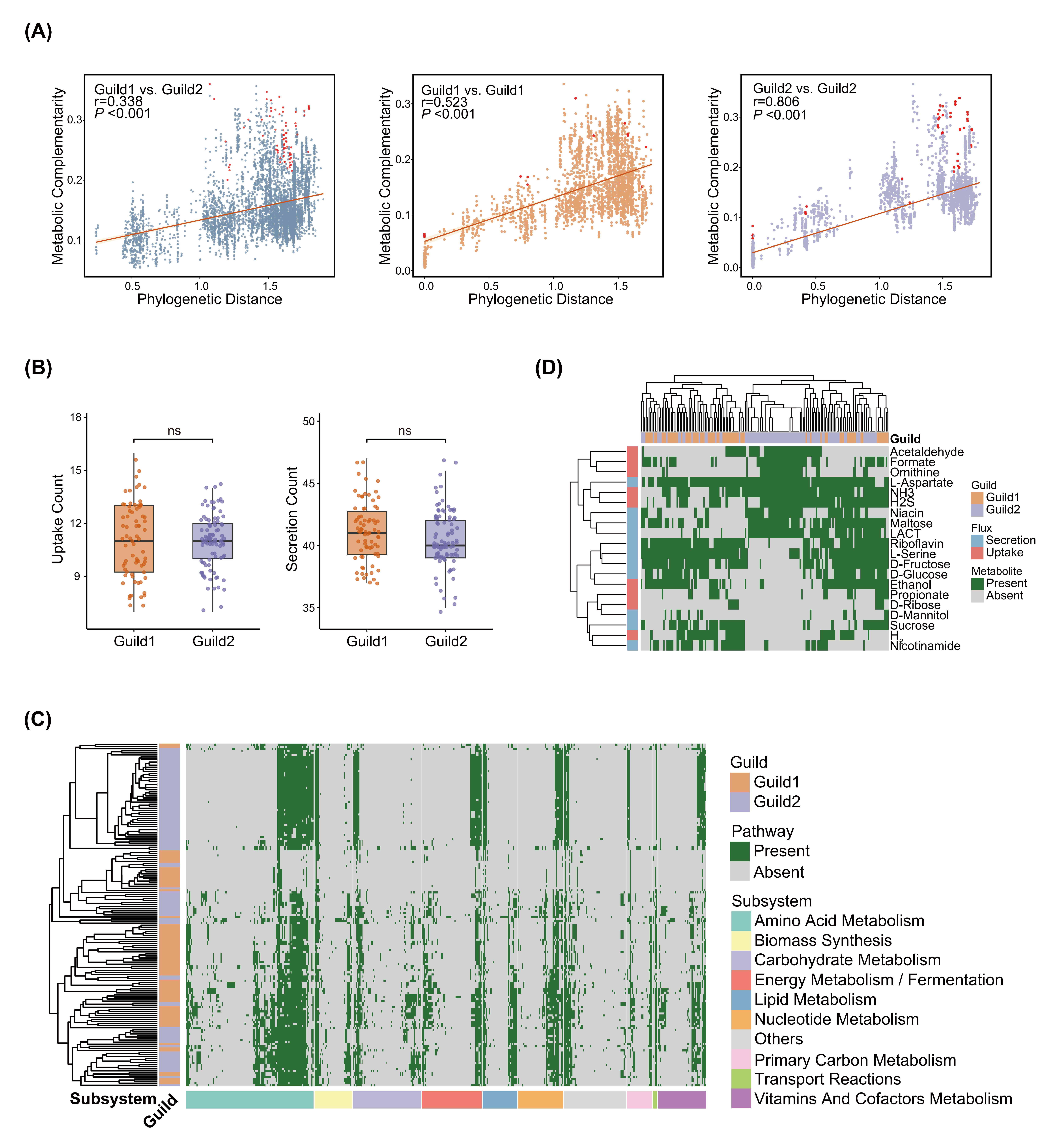

Supplement: Supplementary Figure S7 — Comparison of metabolic indices, metabolic potential, metabolic fluxes, and metabolite exchange between two guilds. (A) The correlations between metabolic complementarity index and phylogenetic distance within Guild 1 and Guild 2, and between Guild 1 and Guild 2. Significant complementary outliers are highlighted in red based on a Z-score threshold of 2.698. (B) Differences between Guild 1 and Guild 2 in the number of metabolites predicted from uptake fluxes (left panel) or secretion fluxes (right panel). P-values were calculated using a two-sided Wilcoxon rank-sum test. ns not significant. (C) Heatmap of metabolic pathway presence-absence across subsystems in HQMAGs from both guilds. (D) Presence-absence patterns of metabolites significantly associated with Guild 1 or Guild 2 in each ADG-associated strain. Columns represent strains colored by guild. Metabolites are clustered by complete-linkage hierarchical clustering based on presence-absence patterns and colored by metabolic flux type, including uptake and secretion. [file Image_7.jpeg]
